# Quality indicators: completeness, validity and timeliness of cancer registry data contributing to the European Cancer Information System

**DOI:** 10.3389/fonc.2023.1219128

**Published:** 2023-07-28

**Authors:** Francesco Giusti, Carmen Martos, Raquel Negrão Carvalho, Liesbet Van Eycken, Otto Visser, Manola Bettio

**Affiliations:** ^1^ European Commission, Joint Research Centre (JRC), Ispra, Italy; ^2^ Belgian Cancer Registry, Brussels, Belgium; ^3^ Rare Diseases Research Unit, Foundation for the Promotion of Health and Biomedical Research in the Valencian Region (FISABIO), Valencia, Spain; ^4^ Department of Registration, Netherlands Comprehensive Cancer Organisation (IKNL), Utrecht, Netherlands

**Keywords:** cancer registry, data quality, completeness, validity, timeliness, Europe

## Abstract

Population-based Cancer Registries (PBCRs) are tasked with collecting high-quality data, important for monitoring cancer burden and its trends, planning and evaluating cancer control activities, clinical and epidemiological research and development of health policies. The main indicators to measure data quality are validity, completeness, comparability and timeliness. The aim of this article is to evaluate the quality of PBCRs data collected in the first ENCR-JRC data call, dated 2015.

**Methods:**

All malignant tumours, except skin non-melanoma, and *in situ* and uncertain behaviour of bladder were obtained from 130 European general PBCRs for patients older than 19 years. Proportion of cases with death certificate only (DCO%), proportion of cases with unknown primary site (PSU%), proportion of microscopically verified cases (MV%), mortality to incidence (M:I) ratio, proportion of cases with unspecified morphology (UM%) and the median of the difference between the registration date and the incidence date were computed by sex, age group, cancer site, period and PBCR.

**Results:**

A total of 28,776,562 cases from 130 PBCRs, operating in 30 European countries were included in the analysis. The quality of incidence data reported by PBCRs has been improving across the study period. Data quality is worse for the oldest age groups and for cancer sites with poor survival. No differences were found between males and females. High variability in data quality was detected across European PBCRs.

**Conclusion:**

the results reported in this paper are to be interpreted as the baseline for monitoring PBCRs data quality indicators in Europe along time.

## 1 Introduction

Population-based Cancer Registries (PBCRs) are tasked with collecting high-quality data, important for monitoring cancer burden and its trends, planning and evaluating cancer control activities, clinical and epidemiological research and developing of health policies (1). Therefore, the value of a PBCR is inherent in the quality of its data and the related quality control measures. The main indicators to measure data quality are validity, completeness, comparability and timeliness (2, 3).

Validity or accuracy refers to the proportion of cases with specific characteristics that actually have such attribute. Completeness indicates the extent of which all incident cancer cases in the area covered by the PBCR are indeed recorded by the PBCR. Comparability is the adherence to common international guidelines. Timeliness refers to how quickly cancer incidence data is collected, processed and reported. There is usually a trade-off between timeliness and both completeness and validity. Cancer data quality indicators include proportion of cases with death certificate only (DCO%), the proportion of microscopically verified cases (MV%) and the mortality to incidence (M:I) ratio (2–4).

The European Network of Cancer Registries (ENCR) has been operating since 1990 to support the collaboration among European PBCRs. One of the ENCR main aims is the improvement of the quality and comparability of cancer incidence data. The ENCR Secretariat has been hosted in Ispra, Italy, since 2012 by the Directorate-General Joint Research Centre (JRC), the science and knowledge centre of the European Commission. The JRC supports the ENCR in the harmonisation of PBCR data, with the goal of accurately comparing data between European areas (5).

In 2015 a first ENCR-JRC data call was launched by the ENCR Steering Committee and the JRC to the European PBCRs (6). After harmonisation, EU-wide statistics on incidence and mortality by cancer site, sex, age group and PBCR have been computed, feeding the European Cancer Information System (ECIS) as the web tool developed and maintained by the JRC to report on the burden of cancer in EU and Europe (7).

The goal of this study is to evaluate the quality of PBCRs data collected in the first ENCR-JRC data call, dated 2015, and is based on indicators evaluating completeness, validity and timeliness as data quality dimensions.

## 2 Methodology

### 2.1 Data sources

**Figure 1:**
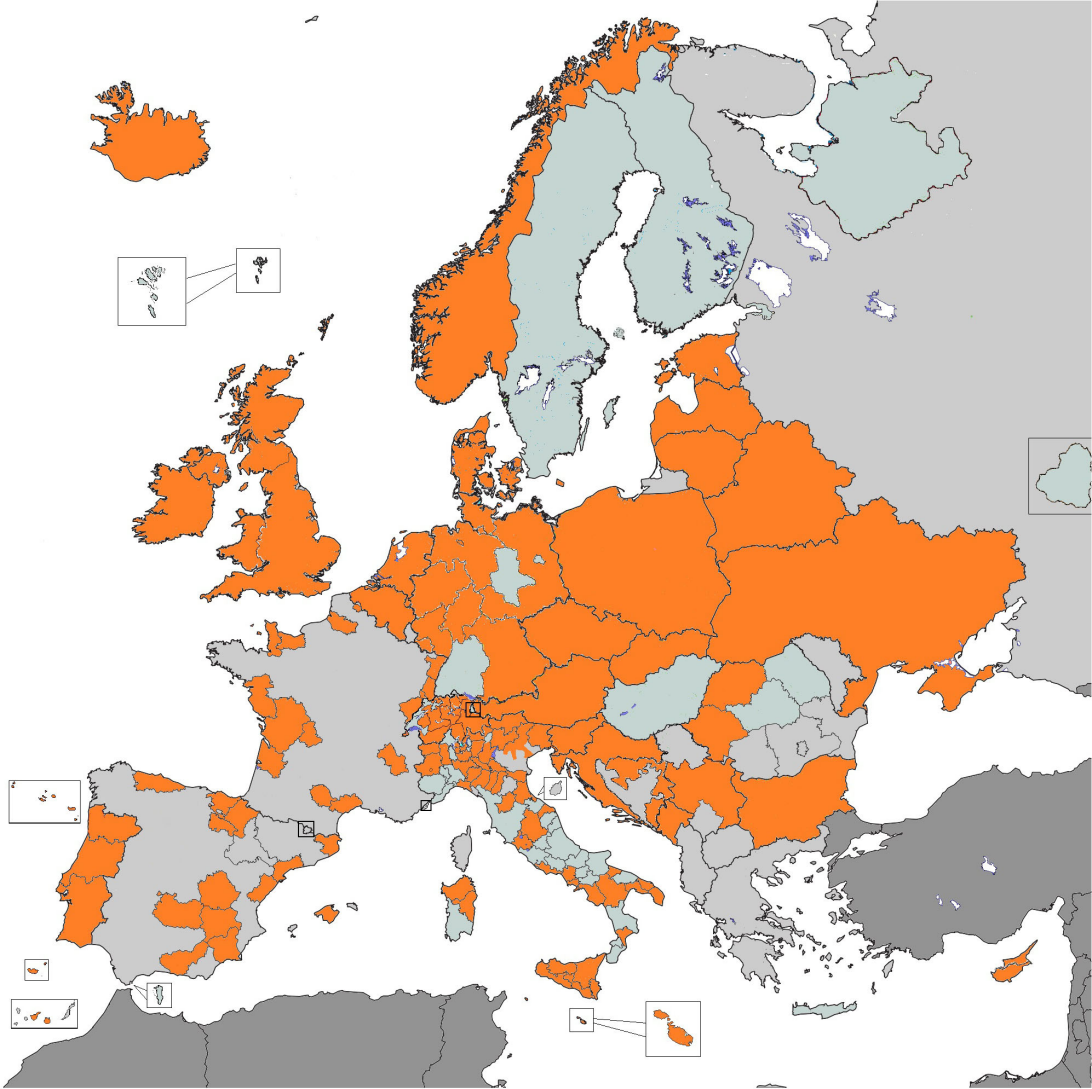
Population-based cancer registries contributing data for the analysis (in orange).

Incidence and mortality data from 130 European general PBCRs (collecting data for all ages and all tumours), contributing to the ECIS through the 2015 ENCR-JRC data call (**Figure 1**; **Supplementary Table 1**) were selected for patients older than 19 years. Data quality in children and adolescents will be analysed in a separate publication, since for this age group tumours are grouped taking into account morphology and topography combinations according the International Classification of Childhood Cancer and also have different definitions of unspecified morphology compared to adults (8).

All malignant tumours (ICD-O-3.1 behaviour = 3), except skin non-melanoma, and *in situ* and uncertain behaviour (ICD-O-3.1 behaviour 2 and 1 respectively) of bladder were included in the analysis.

Among others, the 2015 data call protocol (9) included the following variables: topography, morphology and behaviour, coded according to the International Classification of Diseases for Oncology, Third Edition, (ICD-O-3) (10), as well as basis of diagnosis.

Patients with the same patient identification code and tumour identification code were checked, and excluded from the analysis if other variables such as topography, morphology and behaviour were also duplicated.

Cancer sites were defined with ICD-O-3 topography and morphology combinations reported in **Supplementary Table 2**.

### 2.2 Quality indicators

Validity, completeness, and timeliness of the PBCRs datasets were evaluated. The following indicators were calculated, with type of indicator specified in italics between parentheses (2, 3):

- DCO% (*validity*).
- Proportion of cases with unknown primary site (PSU%, *validity*) (ICD-O-3 topography = C80.9).
- MV% (*validity* and *completeness*). Tumours with basis of diagnosis as cytology, histology of a primary tumour or histology of a metastasis were considered as MV cases.
- M:I ratio (*completeness*), computed dividing the number of deaths by the number of incident cases.
- Proportion of cases with unspecified morphology (UM%, *validity*). The ICD-O-3.1 morphology codes considered as unspecified morphologies were 8000-8005 for solid tumours and 9590-9591, 9596, 9727, 9760, 9800-9801, 9805-9809, 9820, 9832, 9835, 9860, 9960, 9970-9971, 9975, 9989 for haematological malignancies.
- Median of the difference between the registration date and the incidence date (*timeliness*). Date of registration was defined in the 2015 data call protocol as the date in which a cancer case was first recorded in the registry database (9).

When applicable, all indicators were disaggregated by sex, age group (20-59, 60-69, 70-79, 80+ years), cancer site, period (1995-1999, 2000-2004, 2005-2009, 2010-2014) and PBCR.

Benchmarks for the latest available period (2010-2014) were computed for the first tertile (30%) of PBCRs with the higher performance for each indicator. Two-sided 95% confidence intervals were calculated using the Clopper–Pearson method for DCO%, MV%, UM%, PSU%, with a ratio paired t-test for M:I ratio and with the normal approximation method for the timeliness indicator.

## 3 Results

A total of 28,776,562 cases from 130 PBCRs, 21 National and 109 regional PBCRs, operating in 30 European countries were included in the analysis (**Figure 1**).

MV%, PSU% and UM% were computed for all 130 PBCRs, DCO% for 102 PBCRs which had access to death certificate information, M:I ratio for 92 PBCRs with available mortality data, and timeliness for the 49 PBCRs which provided date of registration.

**Table 1** includes DCO%, MV%, UM%, M:I ratio by age at diagnosis and cancer site for the period 1995-2014 and timeliness for the period 2000-2014.

**Table 1:** Proportion of cases: with death certificate only (DCO%), microscopically verified (MV%), with unspecified morphology (UM%), by age at diagnosis and cancer site, 1995-2014.

Cancer site/age	DCO%					MV%					UM%					M:I ratio					Timeliness				
	20-59	60-69	70-79	80+	Total	20-59	60-69	70-79	80+	Total	20-59	60-69	70-79	80+	Total	20-59	60-69	70-79	80+	Total	20-59	60-69	70-79	80+	Total
Lip, Oral cavity and Pharynx	1.3	1.7	2.4	5.2	2.0	96.6	95.7	94.1	88.1	95.0	2.7	3.4	4.5	8.9	3.8	0.32	0.38	0.42	0.55	0.38	610	623	723	730	650
Oesophagus	2.6	2.7	3.1	5.1	3.3	92.8	91.8	89.0	80.5	88.9	5.2	5.5	6.5	10.5	6.7	0.78	0.84	0.93	1.07	0.90	422	392	371	396	394
Stomach	3.2	4.3	5.8	10.7	6.3	92.6	89.9	86.7	76.3	86.0	7.3	9.2	11.1	17.6	11.5	0.60	0.65	0.72	0.89	0.73	671	681	686	709	690
Colon and Rectum	1.2	1.7	2.9	7.4	3.4	95.9	94.4	91.1	78.6	89.9	3.0	4.0	6.1	13.7	6.8	0.33	0.35	0.43	0.65	0.46	628	634	665	668	650
Liver	8.1	9.3	11.3	17.2	11.4	62.1	58.1	51.0	31.3	51.2	19.4	21.0	23.7	31.6	23.7	0.81	0.90	1.01	1.19	0.98	966	1053	1032	1006	1021
Pancreas	5.3	6.7	9.2	15.1	9.4	73.8	65.8	51.8	26.6	52.9	21.2	25.7	34.7	51.0	34.1	0.84	0.92	0.98	1.07	0.97	720	712	761	786	750
Larynx	1.8	2.1	3.2	7.1	2.7	96.0	94.9	93.0	84.4	94.0	3.4	4.0	5.1	10.6	4.6	0.34	0.37	0.46	0.72	0.42	760	790	836	791	790
Lung	3.3	3.9	5.1	9.3	5.2	86.2	81.4	72.9	49.2	74.2	10.3	12.8	17.0	29.7	16.5	0.75	0.81	0.88	1.01	0.86	713	656	690	724	693
Melanoma of the Skin	0.3	0.5	0.8	2.2	0.7	98.2	98.0	97.1	95.1	97.6	–	–	–	–	–	0.13	0.17	0.22	0.32	0.20	418	377	373	347	392
Breast (Female)	0.4	0.9	2.0	7.5	1.8	98.0	97.0	93.9	82.0	94.8	1.6	2.4	4.7	12.2	3.8	0.17	0.23	0.34	0.57	0.28	525	523	578	617	547
Cervix Uteri	0.5	1.7	3.5	8.6	1.6	98.1	95.6	92.4	82.5	95.9	1.7	3.6	5.9	12.1	3.2	0.22	0.39	0.56	0.80	0.34	384	506	534	582	425
Uterus: Corpus and Unspecified	0.5	0.7	1.9	8.2	2.0	98.3	97.9	95.5	82.9	95.4	1.3	1.5	3.1	11.8	3.2	0.13	0.19	0.31	0.60	0.26	670	633	671	722	668
Ovary	1.3	2.5	4.7	12.1	4.1	94.7	91.0	83.5	59.7	85.6	4.4	7.0	12.2	27.7	10.6	0.44	0.64	0.80	0.98	0.70	590	549	579	653	590
Prostate	0.3	0.6	1.9	9.2	2.6	97.7	96.6	91.9	67.8	89.9	1.6	2.2	5.1	19.6	6.3	0.08	0.11	0.24	0.70	0.26	448	560	714	792	633
Testis	0.2	2.0	7.6	17.3	0.5	97.8	94.0	84.9	59.6	97.2	1.4	5.2	13.1	31.0	1.9	0.05	0.16	0.33	0.55	0.06	500	564	707	941	508
Bladder	0.6	0.9	1.8	5.4	2.3	96.7	95.9	94.0	84.9	92.7	2.7	3.2	4.4	10.5	5.3	0.15	0.21	0.31	0.57	0.32	890	877	866	828	866
Kidney, Renal Pelvis, Ureter	1.2	2.0	3.6	9.7	3.4	91.6	86.9	78.1	48.3	79.9	6.1	8.9	14.6	33.1	13.4	0.26	0.34	0.43	0.67	0.41	673	654	721	799	707
Central Nervous System	3.3	5.1	8.5	14.9	6.1	86.0	77.9	56.3	20.5	70.8	9.8	15.1	27.8	52.5	19.4	0.70	0.71	0.72	0.67	0.82	659	669	755	834	712
Thyroid	0.2	1.1	3.1	9.2	1.3	98.1	96.2	92.0	77.6	95.9	1.6	3.0	6.1	16.7	3.2	0.03	0.16	0.36	0.79	0.14	866	837	803	671	851
Hodgkin Lymphoma	0.6	2.4	3.9	8.3	1.6	97.3	94.2	92.1	85.8	95.8	–	–	–	–	–	0.13	0.38	0.55	0.88	0.25	694	618	670	582	672
Non-Hodgkin Lymphoma	1.0	1.6	2.9	5.8	2.5	95.7	94.4	91.6	85.3	92.4	22.3	24.0	28.3	35.3	26.7	0.24	0.36	0.49	0.71	0.42	648	635	669	626	646
Other haematological malignancies (HM)	2.3	3.7	6.4	13.2	6.4	91.5	88.8	84.1	72.9	84.6	12.4	8.7	10.2	14.5	11.3	0.41	0.51	0.67	0.89	0.62	784	827	867	860	834
Mesothelioma	1.5	2.0	2.9	5.7	2.9	93.7	92.6	88.5	78.1	88.6	–	–	–	–	–	0.67	0.79	0.85	0.96	0.84	701	737	716	641	701
Primary site unknown (C80)	4.7	6.6	9.5	16.7	10.5	75.1	64.1	50.9	31.5	51.7	21.9	28.4	35.3	47.4	35.4	0.70	0.83	0.91	1.03	0.90	565	580	650	706	649
Other	1.8	3.4	5.9	12.8	6.0	91.1	87.6	81.7	66.3	81.9	7.5	10.3	15.2	26.7	14.8	0.39	0.56	0.68	0.92	0.64	709	754	812	800	773
Total	1.4	2.3	4.0	9.4	3.9	93.9	90.3	84.2	67.6	85.4	5.8	7.8	11.7	22.3	11.1	0.32	0.44	0.55	0.79	0.51	625	647	715	730	678

Mortality to incidence (M:I) ratio by age at diagnosis and cancer site, 1995-2014. Timeliness by age at diagnosis and cancer site, 2000-2014.

**Table 2** includes reference values based for the best performing tertile of PBCRs for DCO%, MV%, UM%, PSU%, M:I ratio and timeliness, by age at diagnosis and cancer site for the period 2010-2014.

**Table 2:** Quality indicators benchmarks, 2010-2014.

	DCO%	MV%	UM%	PSU%	M:I ratio	Timeliness
**Total**	0.3 (0.3-0.3)	95.6 (95.5-95.6)	5.5 (5.5-5.5)	1.3 (1.3-1.3)	0.41 (0.40-0.41)	336 (237-435)
Sex						
Males	0.3 (0.2-0.3)	95.4 (95.3-95.4)	5.6 (5.6-5.7)	1.3 (1.2-1.3)	0.42 (0.41-0.43)	354 (254-454)
Females	0.3 (0.3-0.3)	95.8 (95.8-95.8)	5.4 (5.4-5.4)	1.4 (1.4-1.4)	0.39 (0.38-0.40)	324 (224-424)
Age group						
20-59	0.1 (0.1-0.1)	98.6 (98.6-98.6)	2.2 (2.1-2.2)	0.8 (0.8-0.9)	0.22 (0.21-0.23)	321 (219-422)
60-69	0.1 (0.1-0.1)	97.8 (97.7-97.8)	2.8 (2.8-2.8)	0.9 (0.9-1.0)	0.32 (0.32-0.33)	325 (225-425)
70-79	0.2 (0.2-0.2)	95.6 (95.5-95.6)	5.2 (5.1-5.2)	1.3 (1.3-1.4)	0.44 (0.43-0.45)	347 (244-449)
80+	1.0 (1.0-1.1)	86.7 (86.6-86.8)	13.8 (13.7-13.9)	2.6 (2.5-2.7)	0.73 (0.71-0.76)	496 (279-714)
Cancer site						
Lip, Oral cavity and Pharynx	0.1 (0.1-0.2)	99.1 (99.0-99.2)	1.2 (1.1-1.2)	–	0.35 (0.32-0.37)	327 (222-433)
Oesophagus	0.3 (0.2-0.4)	98.4 (98.2-98.5)	2.2 (2.1-2.3)	–	0.78 (0.73-0.84)	307 (210-403)
Stomach	0.3 (0.3-0.4)	98.1 (98.0-98.2)	2.6 (2.5-2.8)	–	0.66 (0.63-0.69)	323 (221-426)
Colon and Rectum	0.2 (0.2-0.3)	97.5 (97.4-97.5)	2.9 (2.9-3.0)	–	0.40 (0.39-0.42)	312 (208-416)
Liver	0.8 (0.7-0.9)	65.2 (64.6-65.8)	15.2 (14.8-15.6)	–	0.86 (0.82-0.91)	463 (248-678)
Pancreas	0.8 (0.7-0.9)	76.8 (76.4-77.2)	20.0 (19.8-20.3)	–	0.91 (0.85-0.96)	494 (264-724)
Larynx	0.1 (0.0-0.1)	98.9 (98.8-99.1)	1.3 (1.1-1.4)	–	0.35 (0.32-0.38)	568 (344-791)
Lung	0.4 (0.3-0.4)	91.4 (91.3-91.5)	8.3 (8.2-8.4)	–	0.78 (0.76-0.79)	346 (249-442)
Melanoma of the Skin	0.0 (0.0-0.0)	99.9 (99.8-99.9)	–	–	0.16 (0.14-0.19)	304 (197-411)
Breast (Female)	0.1 (0.1-0.1)	99.4 (99.4-99.5)	0.8 (0.8-0.8)	–	0.21 (0.20-0.22)	296 (195-397)
Cervix Uteri	0.1 (0.0-0.1)	99.5 (99.4-99.6)	0.8 (0.7-0.9)	–	0.26 (0.23-0.29)	423 (196-650)
Uterus: Corpus and Unspecified	0.1 (0.1-0.2)	99.0 (98.9-99.1)	1.2 (1.1-1.3)	–	0.28 (0.25-0.31)	410 (232-587)
Ovary	0.3 (0.3-0.4)	95.7 (95.5-96.0)	5.5 (5.3-5.7)	–	0.69 (0.66-0.73)	339 (236-443)
Prostate	0.2 (0.2-0.2)	97.7 (97.6-97.7)	3.3 (3.2-3.4)	–	0.19 (0.18-0.20)	402 (271-533)
Testis	0.0 (0.0-0.1)	99.5 (99.4-99.7)	1.0 (0.8-1.1)	–	0.03 (0.03-0.04)	430 (211-649)
Bladder	0.1 (0.1-0.2)	98.5 (98.4-98.6)	2.3 (2.2-2.4)	–	0.25 (0.22-0.27)	344 (240-448)
Kidney, Renal Pelvis, Ureter	0.2 (0.2-0.3)	90.1 (89.8-90.3)	8.3 (8.1-8.4)	–	0.33 (0.31-0.35)	436 (262-610)
Central Nervous System	0.3 (0.2-0.4)	84.6 (84.1-85.1)	9.5 (9.2-9.8)	–	0.77 (0.73-0.82)	347 (258-436)
Thyroid	0.1 (0.0-0.1)	99.6 (99.5-99.6)	1.1 (1.0-1.2)	–	0.07 (0.06-0.09)	357 (252-463)
Hodgkin Lymphoma	0.0 (0.0-0.1)	99.8 (99.7-99.9)	–	–	0.15 (0.12-0.18)	463 (248-678)
Non-Hodgkin Lymphoma	0.2 (0.2-0.3)	99.4 (99.3-99.4)	12.8 (12.6-13.0)	–	0.32 (0.30-0.34)	444 (251-636)
Other haematological malignancies (HM)	0.4 (0.4-0.5)	99.1 (99.0-99.2)	6.4 (6.2-6.6)	–	0.62 (0.58-0.65)	552 (343-761)
Mesothelioma	0.2 (0.1-0.4)	99.1 (98.8-99.3)	–	–	0.88 (0.80-0.96)	327 (234-420)
Primary site unknown (C80)	1.4 (1.2-1.5)	76.6 (76.1-77.0)	28.4 (28.0-28.7)	–	0.84 (0.73-0.95)	529 (304-753)
Other	0.5 (0.5-0.6)	94.5 (94.3-94.6)	8.6 (8.5-8.7)	–	0.48 (0.44-0.51)	517 (289-745)

Results by period (**Figures 2**–**6**; **Table 1**; **Supplementary Figures 1, 3, 5, 8**) included PBCRs with available incidence data at least in period 1998-2011.

**Figure 2:**
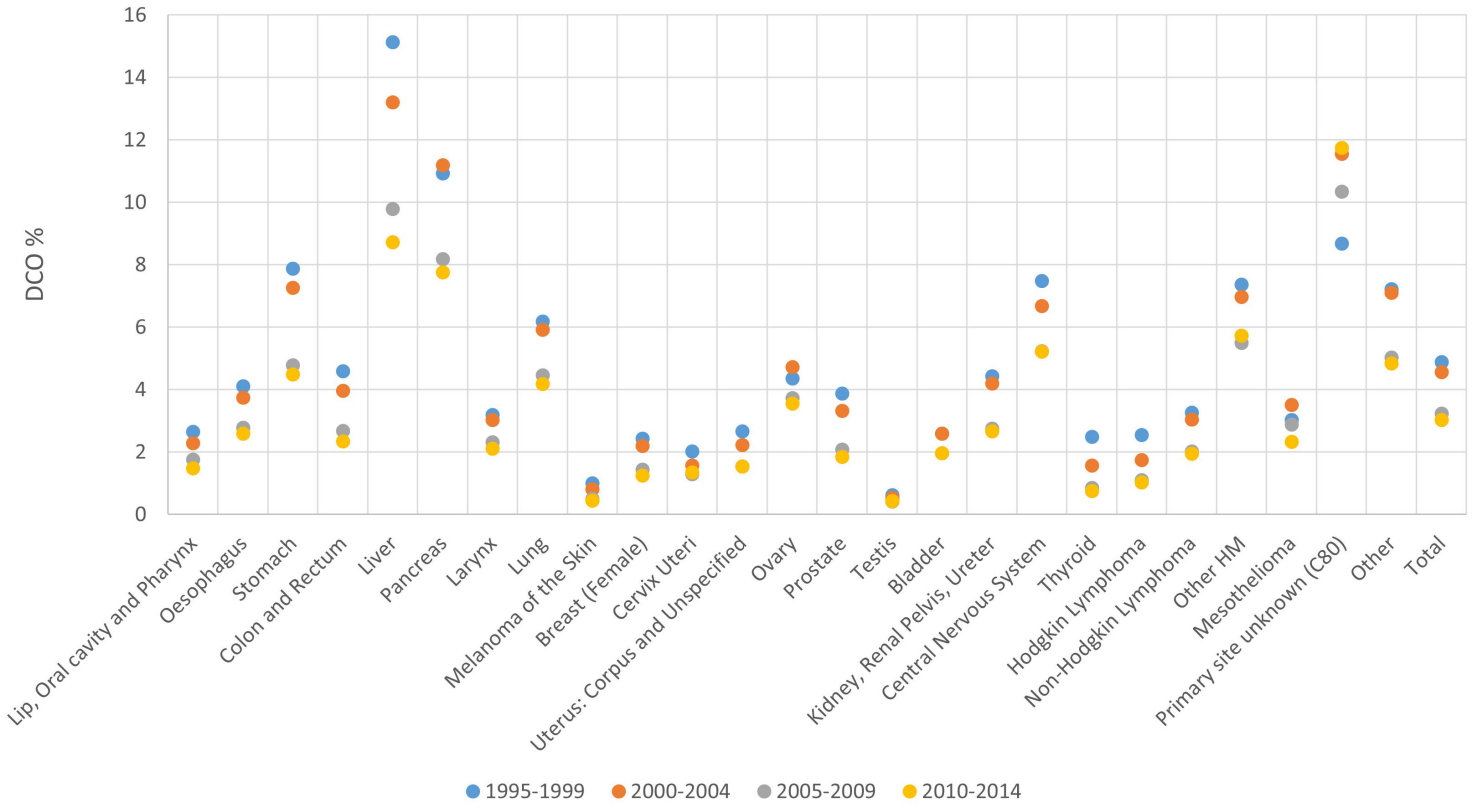
Proportion of cases with death certificate only (DCO%) by period of diagnosis and cancer site, 1995-2014.

**Figure 3:**
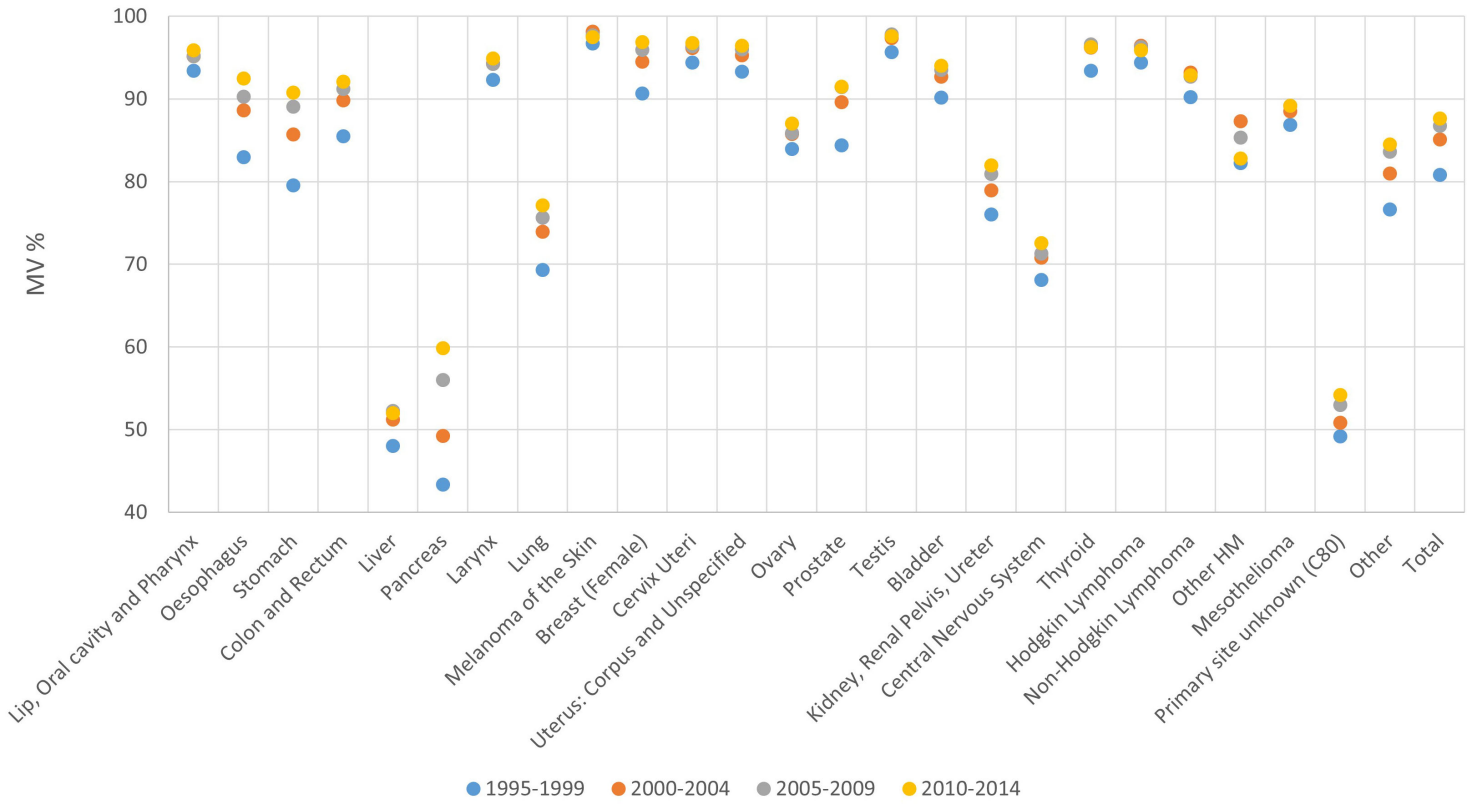
Proportion of microscopically verified cases (MV%) by period of diagnosis and cancer site, 1995-2014.

**Figure 4:**
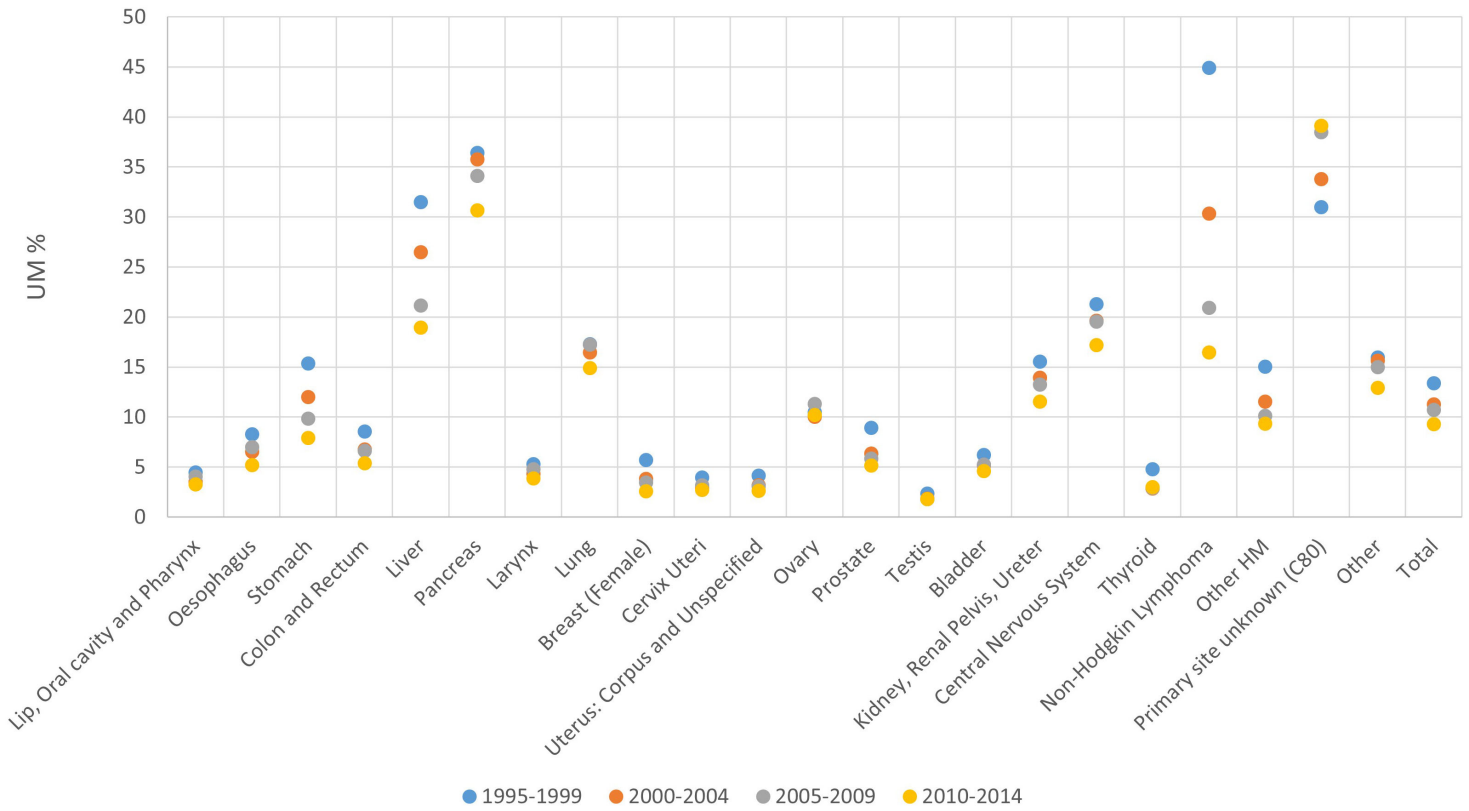
Proportion of cases with unspecified morphology (UM%) by period of diagnosis and cancer site, 1995-2014.

**Figure 5:**
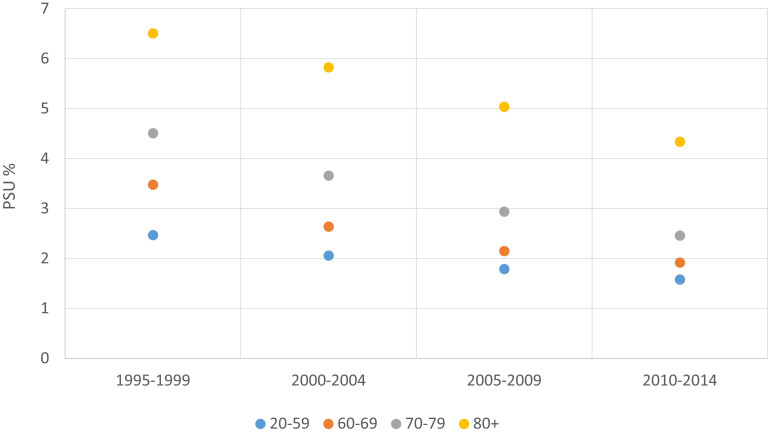
Proportion of cases with unknown primary site/primary site uncertain (PSU%) by age group and period of diagnosis, 1995-2014.

**Figure 6:**
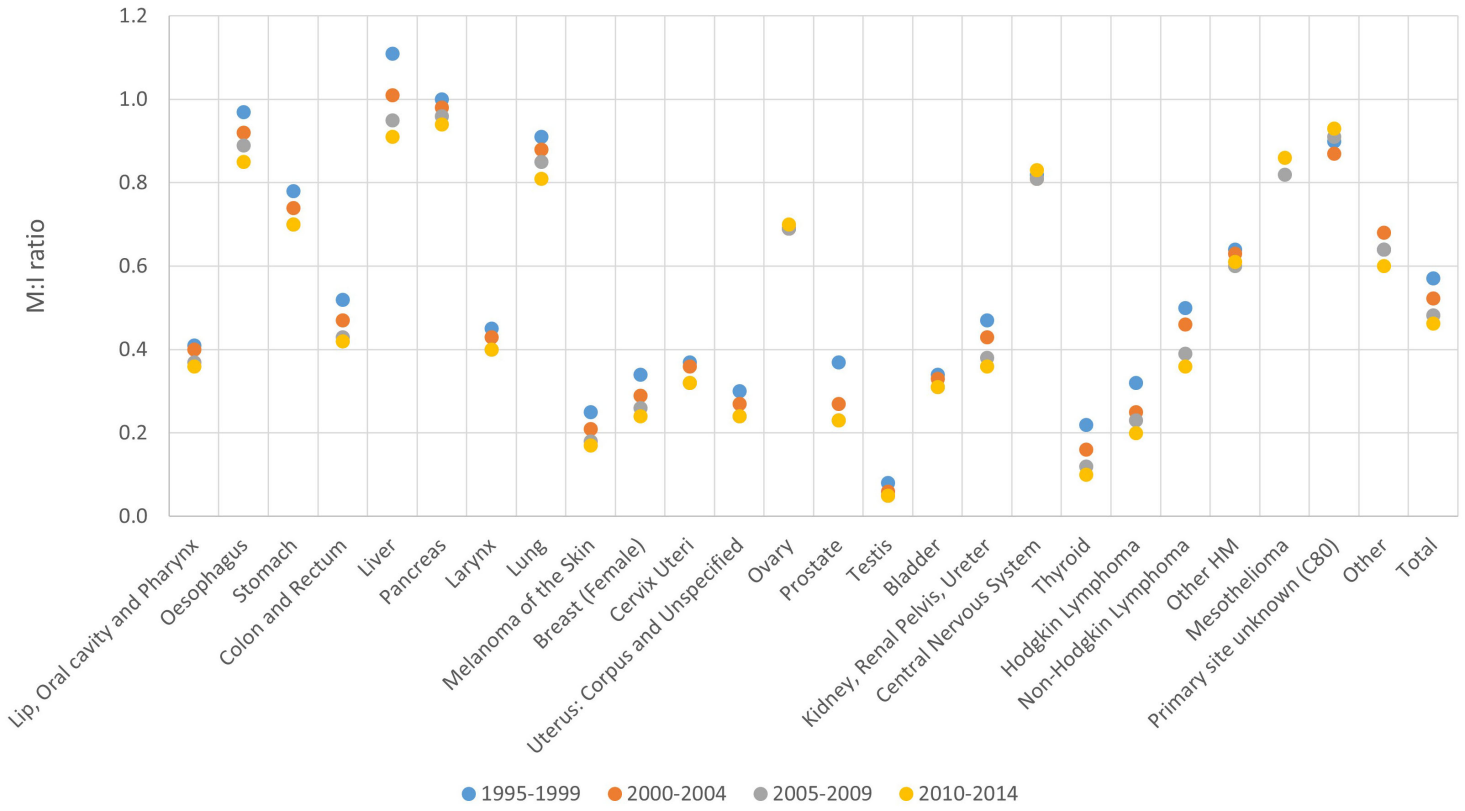
Mortality to incidence (M:I) ratio by period of diagnosis and cancer site, 1995-2014.

**Figure 7:**
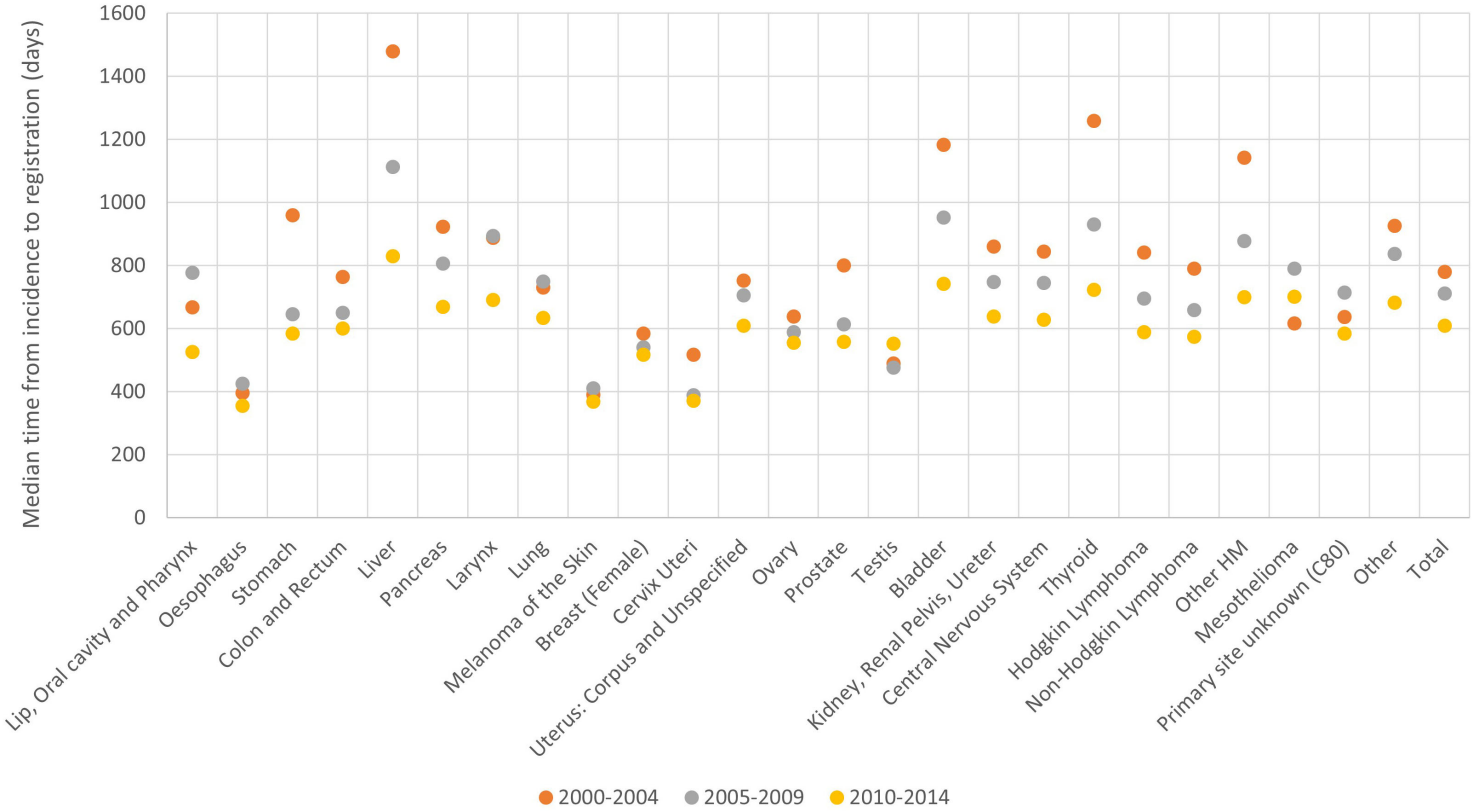
Timeliness by period of diagnosis and cancer site, 2000-2014.

Results by period for timeliness (**Figure 7**; **Table 1**; **Supplementary Figure 10**) included PBCRs with available incidence data at least in period 2003-2011, with at least 2 incidence years in each considered period: 2000-2006 and 2007-2014.

### 3.1 Proportion of cases with death certificate only (DCO%)

The highest DCO% was recorded for liver, pancreas cancer and unknown primary site cases, followed by other haematological malignancies, stomach cancer, brain and central nervous system tumours and lung cancer. The lowest DCO% occurred for testicular cancer, skin melanoma and cervical cancer (**Figure 2**).

When comparing different time periods, a decrease in DCO% was observed over time for all cancer sites, except PSU cases, changing on average from 4.9% in the period 1995-1999 to 3.0% in the period 2010-2014 (**Figure 2**). In particular, between 1995-1999 and 2010-2014 DCO cases decreased on average from 15.1% to 8.7% for liver, from 10.9% to 7.8% for pancreatic cancer and from 7.9% to 4.5% for stomach respectively (**Figure 2**).

The DCO% for all PBCRs and all cancer sites combined did not show any difference between males (3.8%) and females (4.0% - data not shown).

Considering the whole analysed period, an increase in DCO% was observed with increasing age, from 1.4% in patients aged 20-59 years at diagnosis, up to 9.4% for those aged 80 and more. Differences by age group were found for most cancer sites. In particular, age group 20-59 and 80+ had a respective DCO% of 8.1% vs 17.2% for liver, 5.3% vs 15.1% for pancreas, 3.3% vs 14.9% for central nervous system and 1.3% vs 12.1% for ovary (**Table 1**; **Supplementary Figure 1**). There was a high variability among PBCRs for this indicator. Whereas the majority of PBCRs had less than 5% DCO cases between 1995 and 2014, 25 out of 102 PBCRs had more than 5% DCOs in at least one of the considered 5-year periods. However, the latter group of PBCRs showed a general improvement for this indicator between 1995 and 2014 (**Supplementary Figure 2**).

### 3.2 Proportion of microscopically verified cases (MV%)

The lowest MV% occurred for hepatic and pancreatic cancer, followed by lung and central nervous system. The highest MV% was observed for lip and oral cancers, larynx, melanoma, female breast cancer, cancer of the cervix and uterus, testis, thyroid and Hodgkin and non-Hodgkin lymphoma (**Figure 3**).

MV% increased over time across cancer sites, from an average 81% in the period 1995-1999, to 88% for the period 2010-2014. The biggest improvement between 1995-1999 and 2010-2014 was observed for pancreas (43% and 60% respectively), stomach (80% and 91% respectively) and oesophagus (83% and 92% respectively) (**Figure 3**).

The MV% was similar for males and females (85% and 86% respectively - data not shown).

When comparing different age groups, the highest MV% (94%) occurred in the younger age group (20-59 years), followed by age groups 60-69 (90%), 70-79 (84%) and 80+ (68%). MV% decreased by age group for all cancer sites. In particular, age groups 20-59 and 80+ had respective MV%s of 86% vs 20% for central nervous system, 74% vs 27% for pancreas and 86% vs 49% for lung (**Table 1**; **Supplementary Figure 3**).

As for DCO%, a high variability among PBCRs was found, although 117 out of 128 PBCRs had an overall MV% of at least 80% in the latest available period of incidence, MV% increased for most PBCRs between 1995-1999 and 2010-2014 (**Supplementary Figure 4**).

### 3.3 Proportion of cases with unspecified morphology (UM%)

The highest UM% was found for non-Hodgkin lymphoma, mainly in period 1995-2004, primary site unknown, pancreas and liver and the lowest was found for testis, thyroid, uterus and lip, oral cavity and pharynx.

The UM% decreased over time, from an average of 13% in the period 1995-1999 to 9% in the period 2010-2014. The highest decrease was observed for non-Hodgkin lymphoma (45% and 16% in the periods 1995-1999 and 2010-2014 respectively), liver (31% vs 19%) and stomach (15% vs 8%) (**Figure 4**).

The UM% was 11% for both males and females (data not shown).

As for the previous indicators, UM% was lower with increasing age for all cancer sites (on average, 6% and 22% for ages 20-59 and 80+ respectively). In particular, age groups 20-59 and 80+ had a respective UM% of 10% vs 53% for central nervous system, 21% vs 51% for pancreas, 6% vs 33% for kidney, renal pelvis and ureter, and 4% vs 28% for ovary (**Table 1**; **Supplementary Figure 5**).

A high variability in UM% was observed among PBCRs, although 112 out of 130 PBCRs had an overall UM% below 20% in the latest available period of incidence. As for previously considered indicators, an improvement occurred for most PBCRs between incidence years 1995-1999 and 2010-2014 (**Supplementary Figure 6**).

### 3.4 Proportion of cases with unknown primary site/primary site uncertain (PSU%)

The PSU% was 3% for both males and females (data not shown). As far as this dimension is considered, data quality decreased with increasing age (**Figure 5**).

Similarly to the other indicators presented above, PSU% improved over time for all age groups (**Figure 5**).

All 130 PBCRs had less than 5% PSU cases in the latest available period, and the indicator decreased for the majority of PBCRs (**Supplementary Figure 7**).

### 3.5 Mortality to incidence (M:I) ratio

The highest M:I ratio was observed for hepatic and pancreatic cancer, followed by cancer of the oesophagus, lung and stomach. The lowest ratio was observed for testicular cancer, followed by thyroid and melanoma of the skin (**Figure 6**).

Overall M:I ratio was 0.53 for males and 0.49 for females in the analysed period (data not shown).

The overall M:I ratio declined over time, from 0.57 in 1995-1999 to 0.46 in 2010-2014. The biggest improvement was observed for liver, which decreased from 1.11 between 1995-1999 to 0.91 between 2010-2014. A decrease in M:I ratio between 1995-1999 and 2010-2014 was observed also for other cancer sites such as oesophagus (0.97 and 0.85 respectively) and prostate (0.37 and 0.23 respectively) (**Figure 6**).

M:I ratio increased with increasing age, from 0.32 in patients aged 20-59 years to 0.79 in patients aged 80+. M:I ratio increased by age group in all cancer sites except central nervous system. In particular, age groups 20-59 and 80+ had a respective M:I ratio of 0.81 vs 1.19 for liver, 0.78 vs 1.07 for oesophagus, 0.84 vs 1.07 for pancreas and 0.44 vs 0.98 for ovary (**Table 1**; **Supplementary Figure 8**).

Out of 92 PBCRs with available mortality data, 76 had an overall M:I ratio between 0.4 and 0.5 in the latest available period of incidence (**Supplementary Figure 9**).

### 3.6 Timeliness

For the 49 PBCRs with available data, the median time from incidence to registration decreased from 781 to 610 days between incidence years 2000-2004 and 2010-2014 (**Figure 7**). This indicator improved particularly for liver (from 1479 to 830 days respectively), thyroid (from 1259 to 723 days) and bladder (from 1184 to 743 days) and remained relatively low throughout incidence years 2000-2014 for oesophagus, melanoma of the skin and cervix uteri (**Figure 7**).

The median time to registration was lower for younger patients for the majority of cancer sites, for instance, for cervix uteri (384 vs 582 days respectively for age groups 20-59 and 80+ years) and prostate (448 vs 792 days) (**Table 1**; **Supplementary Figure 10**).

A huge variability in timeliness was observed among PBCRs, although 31 out of 49 considered PBCRs had a median time from incidence date to registration date between one and four years in the latest available period. For most PBCRs the indicator improved between incidence years 2000-2004 and 2010-2014 (**Supplementary Figure 11**).

## 4 Discussion

This article gives an overview of data quality among the European PBCRs contributing to the ECIS in the 2015 ENCR-JRC data call. Reference values were computed for the most recently available incidence period (2010-2014) in order to evaluate data quality for future submission to the ECIS (**Table 2**).

Most of the indicators computed in this study have been used at international level for comparing and interpreting cancer data among different PBCRs (2–4, 11). In addition, PBCRs are using them for data quality evaluation (12–21). UM% and PSU% were computed for all 130 PBCRs included in the analysis. UM% and PSU% indicators are based on topography and morphology variables, considered as core variables and available for all PBCRs.

A limitation of this first evaluation is the delay after the latest submissions, in 2018, to the previous ENCR-JRC data call and the present analysis. The benchmarks that were calculated and the experience with the previous data call will help reducing such delay in future data quality assessments in ECIS.

MV% and DCO% were computed on the basis of diagnosis variable, which is also considered a core variable and also available for all PBCRs. Nevertheless, the “death certificate only” category (i.e. basis of diagnosis = 0) of this variable is available only for PBCRs with access to death certificate.

Mortality data by cause of death, sex and age group were not available for 38 PBCRs and M:I ratio could therefore not be computed for these PBCRs.

Only 49 PBCRs submitted registration date for at least two years in each of the two considered periods (2000-2006 and 2007-2014). Therefore, timeliness, median of the difference between the registration date and the incidence date, was computed for the 49 PBCRs.

It will be not possible to compute timeliness at European level in the near future, because date of registration is among the variables not included in the 2022 Call for Data Protocol for European Population-Based Cancer Registries (22) due to the low number of the PBCR that submitted this variable in the 2015 ENCR-JRC data call. Nevertheless, this indicator could be useful at PBCR level for improving the efficiency of PBCR procedures (2).

The use of death certificates as information source is a mean for PBCRs of finding cases not captured by other registration procedures (23). A higher DCO% is often linked to poor cancer prognosis. A high percentage of DCOs can point out incompleteness, as well as low validity.

Liver and pancreas were the cancer sites with the highest proportion of DCO%. This observation is consistent with data from other PBCRs (12, 16, 18). In any case, the DCO% varies highly across cancer sites. The Finnish PBCR reported an overall DCO% (all sites) of 2.6%, also with high differences between cancers. The highest values were reported for unspecified topographies such as respiratory tract NOS (C37 and C39), other digestive organs (C26) and uterus NOS (C55, C58) with values 39%, 23% and 20% respectively. The DCO% for pancreas was 9.5% and for liver 4.8% (16).

A decrease in DCO% was observed between 1995-1999 and 2010-2014. This is in line with what reported for similar periods in Cancer Incidence in Five Continents volumes IX and X (24, 25) and as reported also in selected PBCRs’ studies, namely Zurich and Zug PBCRs, where the proportion of DCO cases declined between 1997 (6.4%) and 2014 (0.8%) (18). As a matter of fact, a declining DCO% trend is a natural consequence of increasing attention and efforts over time to improve data quality. An important activity aimed at improving PBCRs data quality is carried out by the JRC and the ENCR, in the form of training opportunities, the set-up of working groups to draft guidelines provided for data coding, registry visits and most importantly validation of cancer registry data itself (26–30).

Although death certificates are available for the majority of the European PBCRs, there is still a consistent percentage (22%) with problems in accessing death certificates. This issue could have an impact on cancer incidence computation and also survival estimations (31). Nevertheless, DCO% is low for the majority of cancer sites and for the European PBCRs contributing to the ECIS. Therefore, it is unlikely to have significant impact in data comparability among PBCRs, in particular in the latest period of incidence. Lastly, it should be noted that the proportion of death certificate initiated cases (DCI%) is presently not available in ECIS. This indicator can be an important complement to evaluate DCO% but is still not routinely reported by many European PBCRs (3).

The MV% was overall high, with an average value of 85%. The lowest MV% was observed for liver and pancreatic cancer. High overall MV% are consistent with what was reported in selected countries, namely Finland (93%) (16), Norway (94%) (12) and Iceland (96%) (13). Lower MV% values were observed for few PBCRs, consistent with what was reported for instance in Ukraine (78%) and Hungary (58%) (19, 21).

Opposite to the overall value, Iceland reported a low MV% (67%) for liver (13), and Finland reported a MV% of 63% for pancreatic cancer (16).

The highest MV% occurred in the youngest age group and declined with increasing age. This could be explained, at least partially, by a lower diagnostic activity in elderly patients.

An increase in MV% over time was observed, in line with what reported for similar periods in Cancer Incidence in Five Continents volumes IX and X (24, 25). MV% is mainly considered as a measure of validity, but a very high proportion of cases diagnosed by histology or cytology may also suggest that a PBCR is over-reliant on pathology as a source of information and might not detect part of the cases normally diagnosed by other means (2). As an example, the Swiss PBCRs of Zurich and Zug reported a MV% of 62% for 1997, which increased to 81% for 2014 (19).

The UM% was 11% in the observed periods, with a decrease from 13% in 1995-1999 to 9% in 2010-2014. This decrease was highest for non-Hodgkin lymphoma, liver and stomach, at least in part explained by the improvement of the diagnosis techniques for these tumours.

The PSU% was overall around 3%. This indicator decreased for the majority of PBCRs over time.

The PSU% reported by the Iceland PBCR was 1.9% for men and 3.1% for women, while it was 2.2% for both sexes in Norway in 2001-2005. In both countries there was an increase of this percentage with advancing age (12, 13). Differences in the age distribution of men and women populations could partially explain these differences. Nevertheless, differences by sex were not found in our study when all PBCRs were considered together.

Since rare tumours are defined by topography and specific morphology (32), UM% and PSU% could have an important impact in rare cancer incidence computation and data comparability.

The M:I ratio declined over time (from 0.57 in 1995-1999 to 0.46 in 2010-2014), confirming the findings from Cancer Incidence in Five Continents volumes IX and X (24, 25) and reported by selected PBCRs studies: in two Swiss PBCRs, the M:I ratio declined from 0.58 in 1980 to 0.37 in 2014 (18). Bulgaria reported an M:I ratio of 0.5 for males, and 0.4 for females (15). The higher M:I ratio for males observed also in our study (0.53 vs 0.49 for females) is also in line with the usual inverse relationship between this indicator and survival, which is higher in females (3, 25).

M:I ratio can help interpreting cancer incidence in PBCRs, by comparing the indicator with cancer incidence rates. A higher M:I ratio could be associated with lower completeness and incidence rates, which should be interpreted with caution (see for instance the example in **Supplementary Figure 12**). Other factors, such as the quality of death certificates, should be also taken into account into the interpretation of M:I ratio.

As a limitation, mortality data was not available for 38 PBCRs at the moment of the analysis; these were mostly regional registries. In some cases, data was provided by PBCRs in a different format from the one required in the ECIS data call protocol (e.g. less than 18 age classes). Following the analysis most of the problems related to such data were solved, and updated mortality figures can be found in the ECIS web application (7).

Timeliness was evaluated computing the median time from incidence to case registration, which ranged between one and four years for the majority of PBCRs recording this information. This is in line with what reported in a survey performed in 2011, where European PBCRs stated a median time from incidence to data publication (which is related with data registration) of 18 months, with a range between 4 months and 5 years (11). Timeliness indicators have not been frequently reported by PBCRs; however the reduction in time to registration observed in our analysis (with an average decrease of 171 days between 2000-2004 and 2010-2014) has a similar trend to what reported by Norway (from over 525 days in 2001 to 261 days in 2005), whereas Iceland reported a median time from date of diagnosis to registration of 238 days (with a range between 49 and 1445 days) (12, 13). Lastly, an increase in time to registration was observed for 3 PBCRs between 2000-2004 and 2010-2014; this could possibly be due to resource constraints, which have been common for smaller regional PBCRs throughout Europe in recent years.

Indicators for European PBCRs such as MV%, DCO% and M:I ratio were found to be similar to those reported for other developed areas worldwide, in particular to North America, Australia and New Zealand (24).

## 5 Conclusion and way forward

The quality of incidence data reported by PBCRs has been improving across the study period. Data quality is worse for the oldest age groups and for cancer sites with poor survival. No differences were found between males and females. High variability in data quality could be detected across European PBCRs.

The harmonisation of PBCR’ data as the input source for the assessment of cancer burden is one of the main aims of the support provided by the JRC to the ENCR to strengthen the basis for monitoring the cancer burden. In order to improve data quality and harmonisation, the JRC and the ENCR have been carrying out several activities along the years, namely the set-up of yearly training agendas and organisation of trainings, the coordination of thematic Working Groups to draft guidelines and recommendations on data coding, the development and provision of common rules and related validation software to check data compliance to agreed EU-wide standards.

In this context, the results reported in this paper are to be interpreted as the baseline for monitoring PBCRs data quality indicators in Europe along time.

## Data availability statement

The data analyzed in this study is subject to the following licenses/restrictions: Requests to access the datasets should be directed to francescogiusti@hotmail.com.

## Ethics statement

Ethical review and approval was not required for the study on human participants in accordance with the local legislation and institutional requirements. Written informed consent for participation was not required for this study in accordance with the national legislation and the institutional requirements.

## Author contributions

The first draft of the manuscript was written by FG and CM. MB supervised data acquisition. All authors contributed to the article and approved the submitted version.
