# Supplementary material for: Quality indicators: completeness, validity and timeliness of cancer registry data contributing to the European Cancer Information System
Source: Front Oncol. 2023 Jul 28;13:1219128. doi: 10.3389/fonc.2023.1219128 (PMC10421659; doi:10.3389/fonc.2023.1219128)
Supplement: Supplementary file 1 [file DataSheet_1.pdf]

# *Supplementary Material*

## *Quality indicators: completeness, validity and timeliness of cancer registry data contributing to the European Cancer Information System (ECIS).*

**Francesco Giusti[1,2*], Carmen Martos[1,3], Manola Bettio[1*], Raquel Negrão Carvalho[1], Liesbet Van Eycken[2], Otto Visser[4]**

[1]European Commission, Directorate General Joint Research Centre, Directorate F-Health and Food, Ispra (VA), Italy

[2]Belgian Cancer Registry, Brussels, Belgium

[3]Foundation for the Promotion of Health and Biomedical Research of Valencia Region (FISABIO), Valencia (Spain)

[4]Department of Registration, Netherlands Comprehensive Cancer Organisation (IKNL), Utrecht, The Netherlands

*** Correspondence:**

Dr. Francesco Giusti, PhD
francescogiusti@hotmail.com

Manola Bettio
JRC-ENCR@ec.europa.eu

| Country | Cancer Registry | Time period covered |
|---|---|---|
| Austria | National Cancer Registry of Austria | 1995-2012 |
| Belarus | National Cancer Registry of Belarus | 1995-2013 |
| Belgium | National Cancer Registry of Belgium | 2004-2013 |
| Bosnia and Herzegovina | Cancer Registry of Republic of Srpska | 2008-2012 |
| Bulgaria | National Cancer Registry of Bulgaria | 1995-2013 |
| Croatia | National Cancer Registry of Croatia | 2000-2012 |
| Cyprus | Cyprus Cancer Registry | 1998-2014 |
| Cyprus | North Cyprus Cancer Registry | 2012-2012 |
| Czechia | National Cancer Registry of Czechia | 1995-2013 |
| Denmark | National Cancer Registry of Denmark | 1995-2014 |
| Estonia | National Cancer Registry of Estonia | 1995-2012 |
| France | Cancer Registry of Bas-Rhin | 1995-2011 |
| France | Cancer Registry Calvados | 1995-2013 |
| France | Cancer Registry of the Doubs | 1995-2013 |
| France | Cancer Registry of Gironde | 2005-2013 |
| France | Cancer Registry of Hérault | 1995-2013 |
| France | Cancer Registry of Haut-Rhin | 1995-2013 |
| France | Cancer Registry of Isère | 1995-2013 |
| France | Cancer Registry of Lille Area | 2008-2012 |
| France | Cancer Registry of Limousin | 2009-2012 |
| France | Cancer Registry of Loire-Atlantique/Vendée | 1998-2013 |
| France | Cancer Registry of Manche | 1995-2013 |
| France | Cancer Registry of Poitou-Charentes | 2008-2013 |
| France | Cancer Registry of Somme | 1995-2013 |
| France | Cancer Registry of Tarn | 1995-2013 |
| Germany | Cancer Registry of Hessen | 2008-2013 |
| Germany | Cancer Registry of Bavaria | 2003-2012 |
| Germany | Common Cancer Registry of the Federal States Berlin, Brandenburg Mecklenburg-Vorpommern Sachsen-Anhalt and the Free States Saxony and Thuringia | 1998-2013 |
| Germany | Cancer Registry of Bremen | 2000-2013 |
| Germany | Cancer Registry of Hamburg | 1997-2012 |
| Germany | Cancer Registry of North Rhine-Westphalia | 1997-2013 |
| Germany | Cancer Registry of Rhineland-Palatinate | 1998-2012 |
| Germany | Cancer Registry of Saarland | 1997-2012 |
| Germany | Cancer Registry of Lower Saxony | 2003-2012 |
| Germany | Cancer Registry of Schleswig-Holstein | 1998-2012 |

| | | |
|---|---|---|
| Iceland | National Cancer Registry of Iceland | 1995-2014 |
| Ireland | National Cancer Registry Ireland | 1995-2012 |
| Italy | Cancer Registry of Aosta Valley | 2007-2012 |
| Italy | Cancer Registry of Basilicata | 2006-2010 |
| Italy | Cancer Registry of Bergamo | 2007-2012 |
| Italy | Cancer Registry of Brindisi | 2006-2008 |
| Italy | Cancer Registry of Caserta | 2008-2010 |
| Italy | Cancer Registry of Cremona | 2005-2010 |
| Italy | Cancer Registry of Pavia Province | 2008-2010 |
| Italy | Cancer Registry of Piacenza | 2006-2014 |
| Italy | Cancer Registry of South Tyrol | 1995-2010 |
| Italy | Cancer Registry of Puglia - Province of Barletta-Andria-Trani | 2006-2012 |
| Italy | Cancer Registry of the Province of Biella and Vercelli | 2008-2012 |
| Italy | Cancer Registry of Brescia | 1999-2010 |
| Italy | Integrated Cancer Registry of Catania-Messina-Siracusa-Enna | 2003-2013 |
| Italy | Cancer Registry of Catanzaro | 2003-2010 |
| Italy | Cancer Registry of Como Province | 2003-2011 |
| Italy | Cancer Registry of Reggio Emilia | 1996-2014 |
| Italy | Cancer Registry of Ferrara Province | 1995-2011 |
| Italy | Cancer Registry of Friuli Venezia Giulia | 1995-2010 |
| Italy | Cancer Registry of Liguria Region | 1995-2010 |
| Italy | Cancer Registry of Latina Province | 1995-2012 |
| Italy | Cancer Registry of Puglia - Province of Lecce | 2003-2008 |
| Italy | Cancer Registry of Lodi | 2003-2012 |
| Italy | Cancer Registry of Macerata Province | 1995-2001 |
| Italy | Cancer Registry of Mantova | 1999-2010 |
| Italy | Cancer Registry of Monza and Brianza | 2007-2012 |
| Italy | Cancer Registry of Milan | 2008-2012 |
| Italy | Cancer Registry of Modena | 1995-2013 |
| Italy | Cancer Registry of the Campania Region | 1996-2013 |
| Italy | Cancer Registry of Nuoro | 2003-2012 |
| Italy | Cancer Registry of Palermo and Province | 2003-2013 |
| Italy | Cancer Registry of Parma | 1995-2014 |
| Italy | Cancer Registry of Ragusa | 1995-2012 |
| Italy | Cancer Registry of Romagna | 1995-2014 |
| Italy | Cancer Registry of Salerno Province | 1996-2010 |
| Italy | Cancer Registry of Sassari Province | 1995-2011 |
| Italy | Cancer Registry of Sondrio Province | 1998-2013 |
| Italy | Cancer Registry of the Province of Syracuse | 1999-2012 |
| Italy | Cancer registry of Puglia - Province of Taranto | 2006-2012 |

| | | |
|---|---|---|
| Italy | Cancer Registry of Trento | 1995-2010 |
| Italy | Cancer Registry of Trapani | 2002-2010 |
| Italy | Cancer Registry of Piedmont Torino City | 2008-2012 |
| Italy | Cancer Registry of Tuscany Region | 1995-2010 |
| Italy | Cancer Registry of Umbria | 1995-2013 |
| Italy | Cancer Registry of Varese | 1995-2012 |
| Italy | Cancer Registry of Veneto | 1995-2010 |
| Italy | Cancer Registry of Viterbo | 2006-2010 |
| Latvia | National Cancer Register of Latvia | 2000-2013 |
| Lithuania | National Cancer Registry of Lithuania | 1995-2012 |
| Malta | National Cancer Registry of Malta | 1995-2013 |
| Montenegro | Registry of Malignant Neoplasms of Montenegro | 2013-2013 |
| Netherlands | National Cancer Registry of Netherlands | 1995-2013 |
| Norway | National Cancer Registry of Norway | 1995-2013 |
| Poland | National Cancer Registry of Poland | 1999-2013 |
| Portugal | Cancer Registry of Azores | 1997-2011 |
| Portugal | Cancer Registry of Portugal Central Region | 2008-2010 |
| Portugal | Cancer Registry of Portugal North Region | 2000-2010 |
| Portugal | Cancer Registry of Portugal South Region | 2000-2013 |
| Romania | Cancer Registry of Cluj | 2008-2011 |
| Romania | Cancer Registry of Timisoara | 2008-2012 |
| Serbia | Cancer Registry of Central Serbia | 1999-2007 |
| Slovakia | National Cancer Registry of Slovakia | 1995-2010 |
| Slovenia | National Cancer Registry of Republic of Slovenia | 1995-2012 |
| Spain | Cancer Registry of Albacete | 2008-2010 |
| Spain | Cancer Registry of Asturias | 2008-2010 |
| Spain | Cancer Registry of Balearic Islands | 1995-2011 |
| Spain | Cancer Registry of Basque Country | 1995-2012 |
| Spain | Cancer Registry of Canary Islands | 1996-2011 |
| Spain | Cancer Registry of Castellón | 2004-2014 |
| Spain | Cancer Registry of Ciudad Real | 2008-2012 |
| Spain | Cancer Registry of Cuenca | 2008-2012 |
| Spain | Cancer Registry of Girona | 1995-2014 |
| Spain | Cancer Registry of Granada | 1995-2012 |
| Spain | Cancer Registry of Murcia | 1995-2010 |
| Spain | Cancer Registry of Navarra | 1995-2010 |
| Spain | Cancer Registry of La Rioja | 1995-2012 |
| Spain | Cancer Registry of Tarragona | 1995-2011 |
| Switzerland | Cancer Registry of Aargau | 2013-2013 |
| Switzerland | Cancer Registry of Bern | 2014-2014 |
| Switzerland | Cancer Registry of Zug | 2011-2013 |

| | | |
|---|---|---|
| Switzerland | Cancer Registry of Zürich | 1995-2013 |
| Switzerland | Cancer Registry of Basel | 1995-2011 |
| Switzerland | Cancer Registry of Fribourg | 2006-2013 |
| Switzerland | Cancer Registry of Geneva | 1995-2013 |
| Switzerland | Cancer Registry of Graubünden and Glarus | 1995-2013 |
| Switzerland | Cancer Registry of St. Gallen-Appenzell | 1995-2013 |
| Switzerland | Cancer Registry of Thurgau | 2012-2013 |
| Switzerland | Cancer Registry of Ticino | 2000-2012 |
| Switzerland | Cancer Registry of Valais | 1995-2014 |
| Ukraine | National Cancer Registry of Ukraine | 2002-2012 |
| United Kingdom | Cancer Registry of England | 1995-2013 |
| United Kingdom | Cancer Registry of Northern Ireland | 1995-2013 |
| United Kingdom | Cancer Registry of Scotland | 1995-2013 |
| United Kingdom | Cancer Registry of Wales | 1995-2012 |

Supplementary Table 1. List of Population-based Cancer Registries (PBCRs) and time period of data contributing to the analysis.

| Cancer site | ICD-O-3 topography | ICD-O-3 morphology |
|---|---|---|
| Lip, Oral cavity and Pharynx | C000-C148 | excluding 9050-9055, 9140, 9590-9992 |
| Oesophagus | C150-C159 | excluding 9050-9055, 9140, 9590-9992 |
| Stomach | C160-C169 | excluding 9050-9055, 9140, 9590-9992 |
| Colon and Rectum | C180-C209 | excluding 9050-9055, 9140, 9590-9992 |
| Liver | C220-C221 | excluding 9050-9055, 9140, 9590-9992 |
| Pancreas | C250-C259 | excluding 9050-9055, 9140, 9590-9992 |
| Larynx | C320-C329 | excluding 9050-9055, 9140, 9590-9992 |
| Lung | C340-C349 | excluding 9050-9055, 9140, 9590-9992 |
| Melanoma of the Skin | C440-C449 | 8720-8790 |
| Breast (Female) | C500-C509 | excluding 9050-9055, 9140, 9590-9992 |
| Cervix Uteri | C530-C539 | excluding 9050-9055, 9140, 9590-9992 |
| Uterus: Corpus and Unspecified | C540-C559 | excluding 9050-9055, 9140, 9590-9992 |
| Ovary | C569 | excluding 9050-9055, 9140, 9590-9992 |
| Prostate | C619 | excluding 9050-9055, 9140, 9590-9992 |
| Testis | C620-C629 | excluding 9050-9055, 9140, 9590-9992 |
| Bladder | C670-C679 | excluding 9050-9055, 9140, 9590-9992 |
| Kidney, Renal Pelvis, Ureter | C649-C669 | excluding 9050-9055, 9140, 9590-9992 |
| Central Nervous System | C700-C729 | excluding 9050-9055, 9140, 9590-9992 |
| Thyroid | C739 | excluding 9050-9055, 9140, 9590-9992 |
| Hodgkin Lymphoma | - | 9650-9667 |
| Non-Hodgkin Lymphoma | - | 9590-9597, 9670-9671, 9673, 9675, 9678-9680, 9684, 9687-9691, 9695, 9698-9702, 9705, 9708-9709, 9712, 9714-9719, 9724-9729, 9734-9735, 9737-9738 |
| Other haematological malignancies (HM) | - | 9733, 9742, 9800-9801, 9805-9809, 9811-9818, 9820, 9823, 9826-9827, 9831-9837, 9840, 9860-9861, 9863, 9865-9867, 9869-9876, 9891, 9895-9898, 9910-9911, 9920, 9930-9931, 9940, 9945-9946, 9948, 9963-9964 |
| Mesothelioma | - | 9050-9055 |
| Primary site unknown (C80) | C809 | excluding 9050-9055 |
| Other | C170-C179, C210-C218, C239-C249, C260-C269, C309-C319, C339, C379-C424, C470-C499, C510-C529, C570-C609, C630-C639, C680-C699, C740-C779 | excluding 9050-9055, 9140, 9590-9992 |

Supplementary Table 2. Definition of cancer sites.

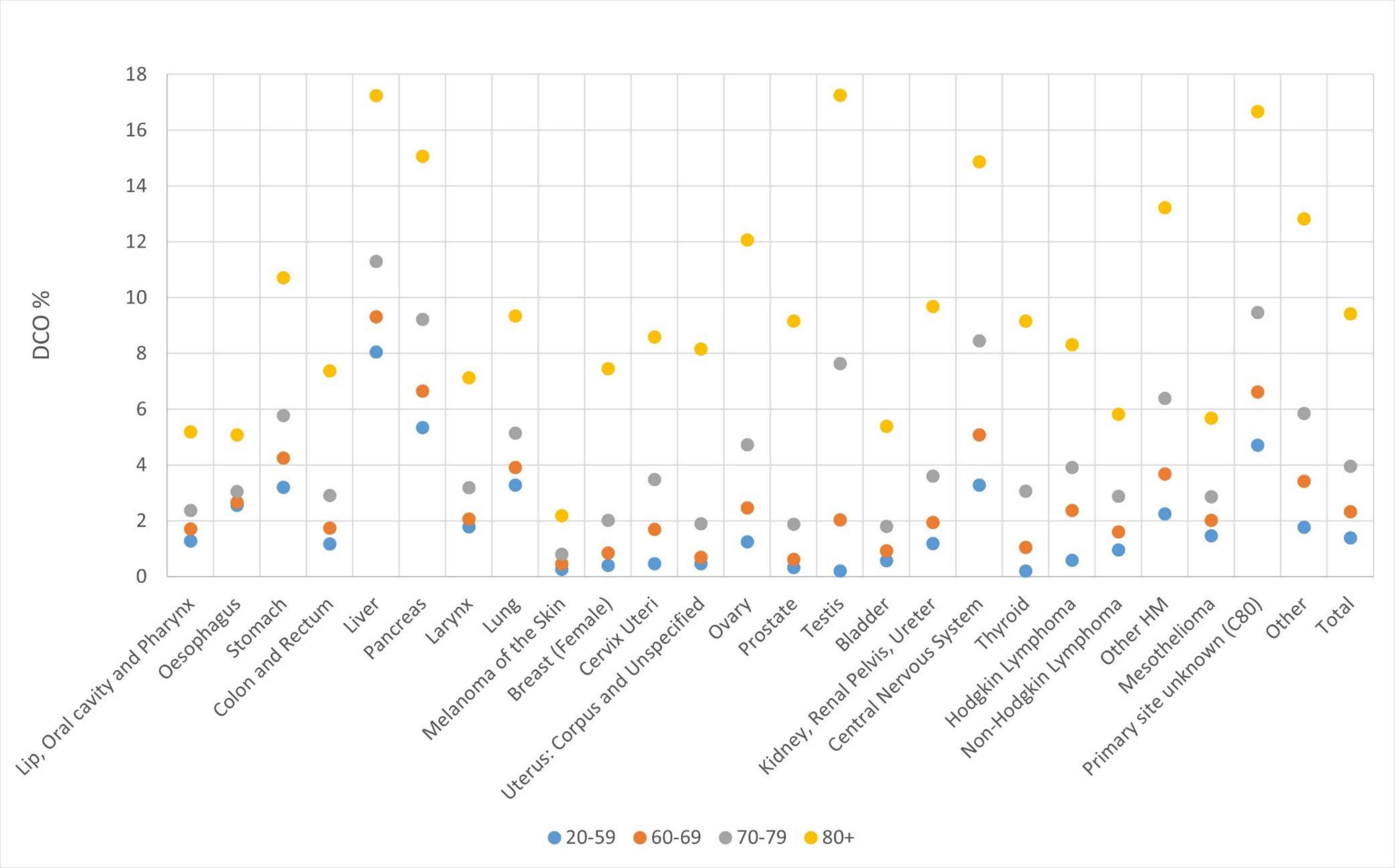


Supplementary Figure 1. Proportion of cases with death certificate only (DCO%) by age at diagnosis and cancer entity, 1995-2014

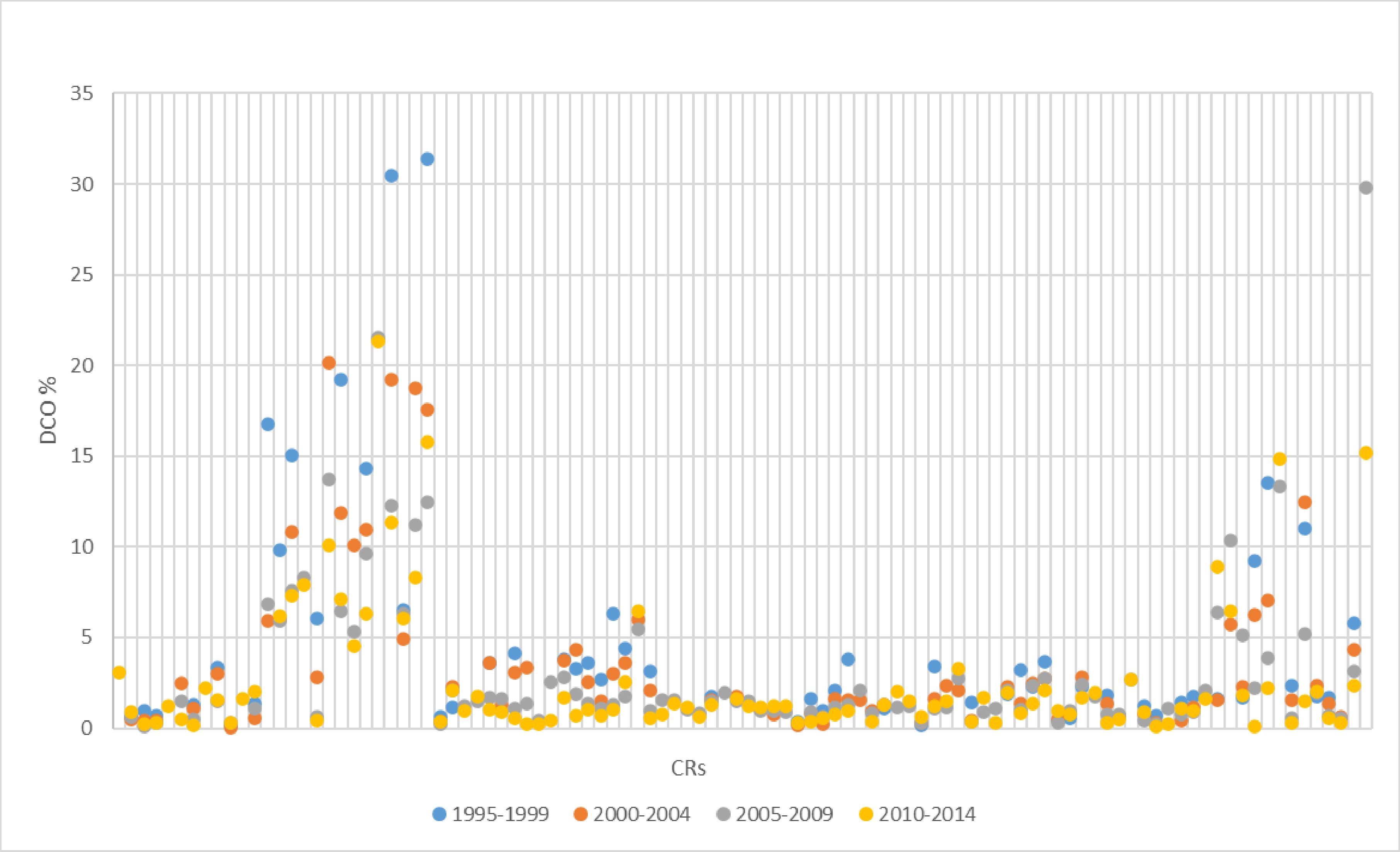


Supplementary Figure 2. Proportion of cases with death certificate only (DCO%) by period of diagnosis and PBCR, 1995-2014

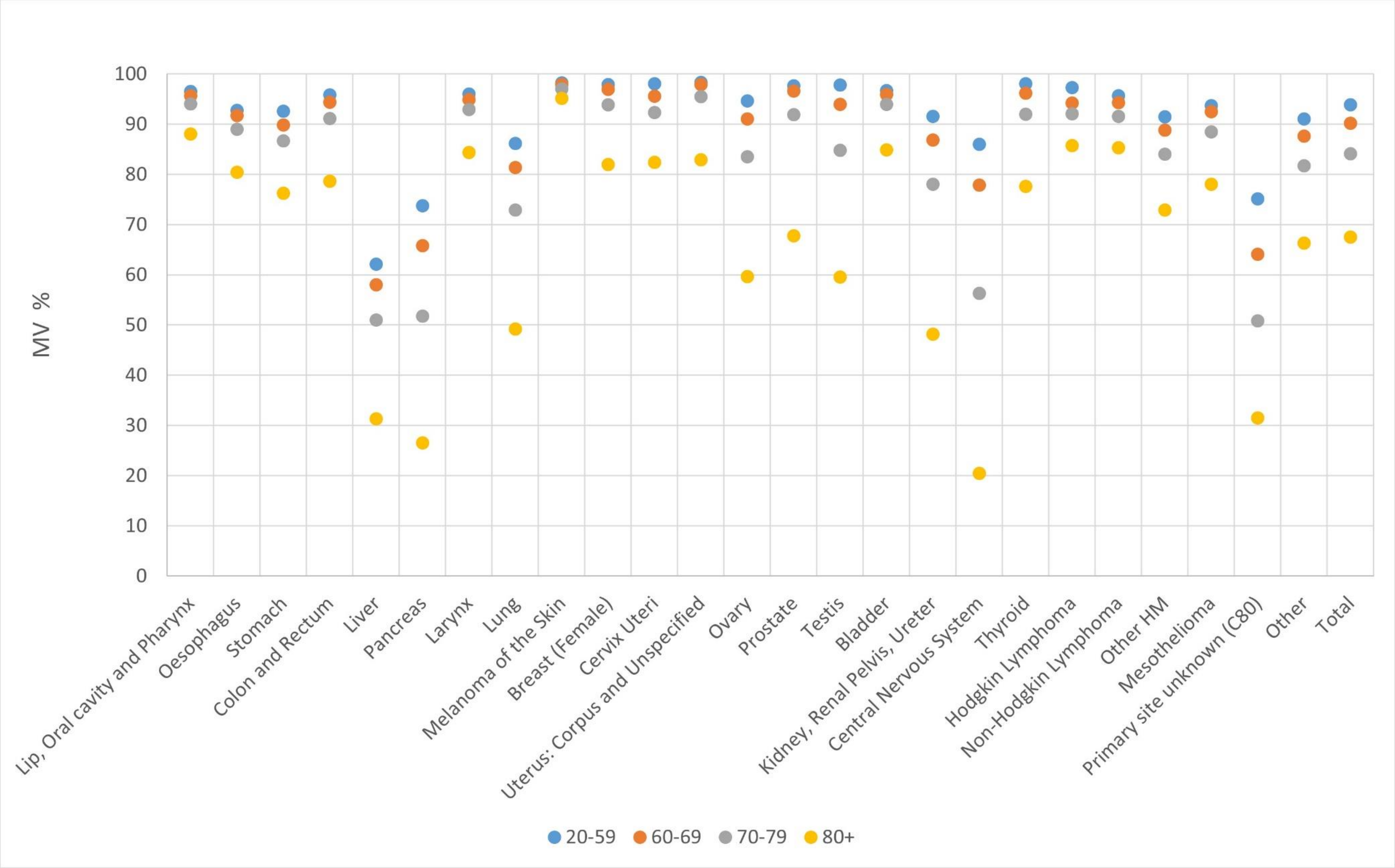


Supplementary Figure 3. Proportion of microscopically verified cases (MV%) by age at diagnosis and cancer entity, 1995-2014

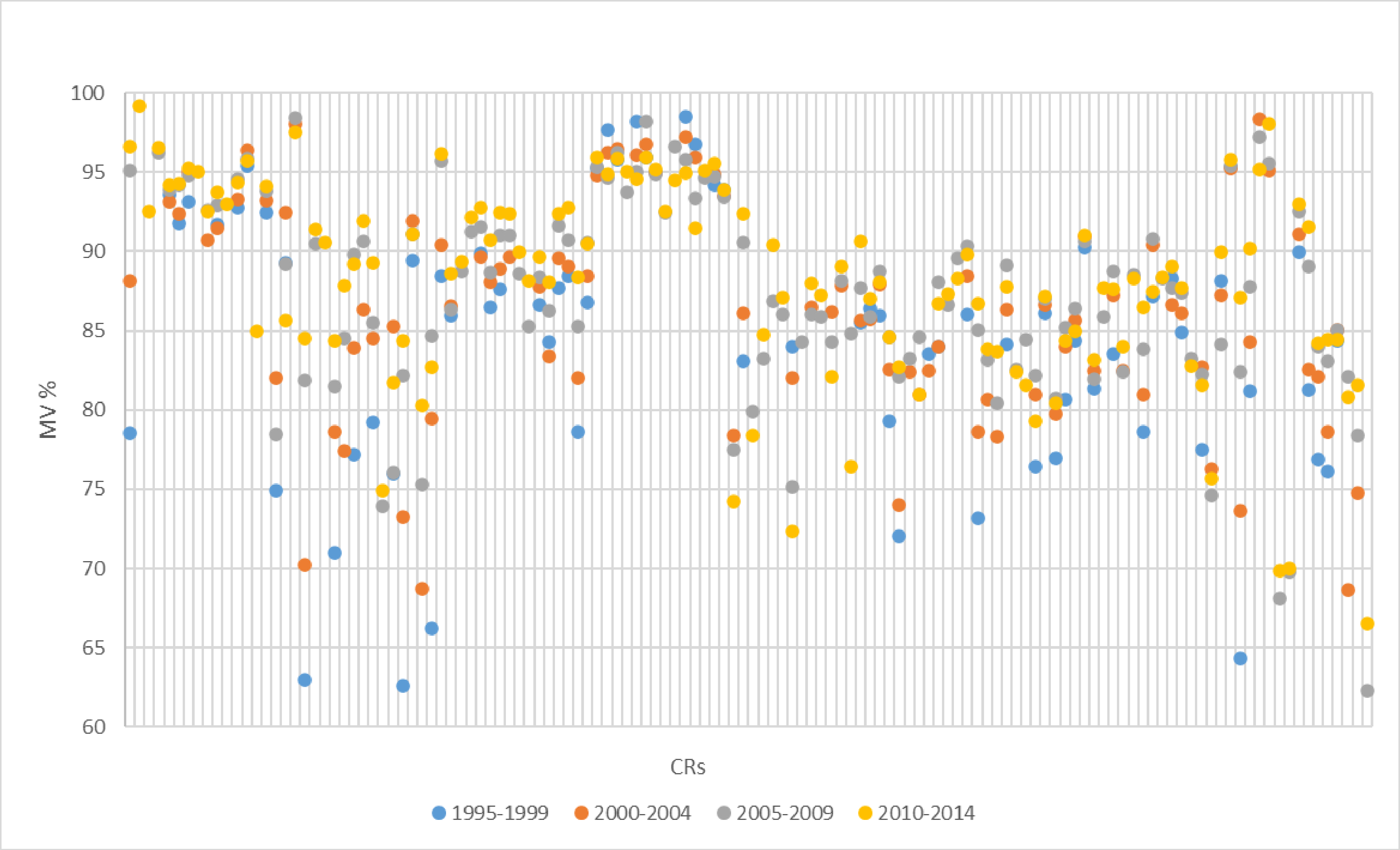


Supplementary Figure 4. Proportion of microscopically verified cases (MV%) by period of diagnosis and PBCR, 1995-2014

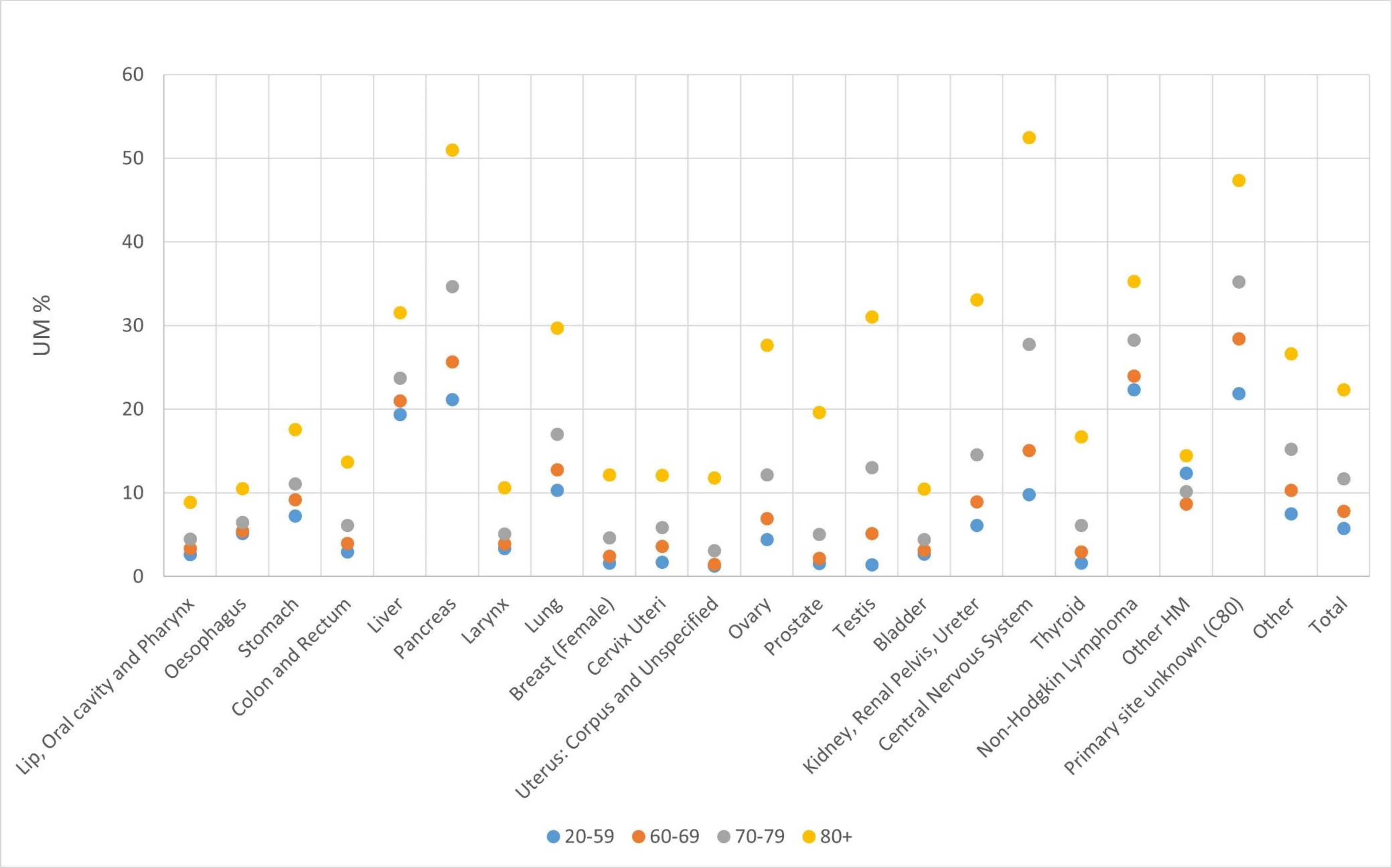


Supplementary Figure 5. Proportion of cases with unspecified morphology (UM%) by age at diagnosis and cancer entity, 1995-2014

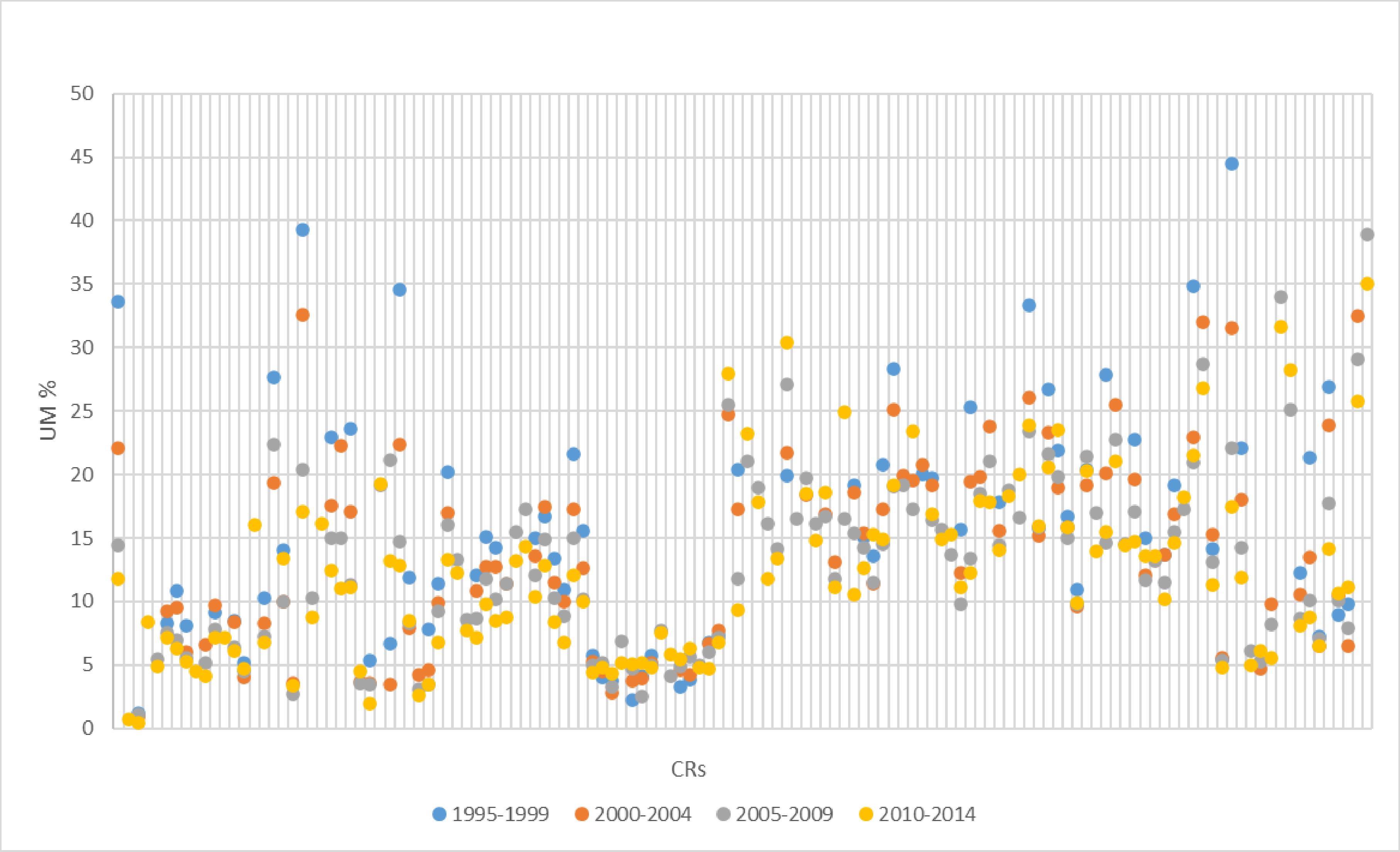


Supplementary Figure 6. Proportion of cases with unspecified morphology (UM%) by period of diagnosis and PBCR, 1995-2014

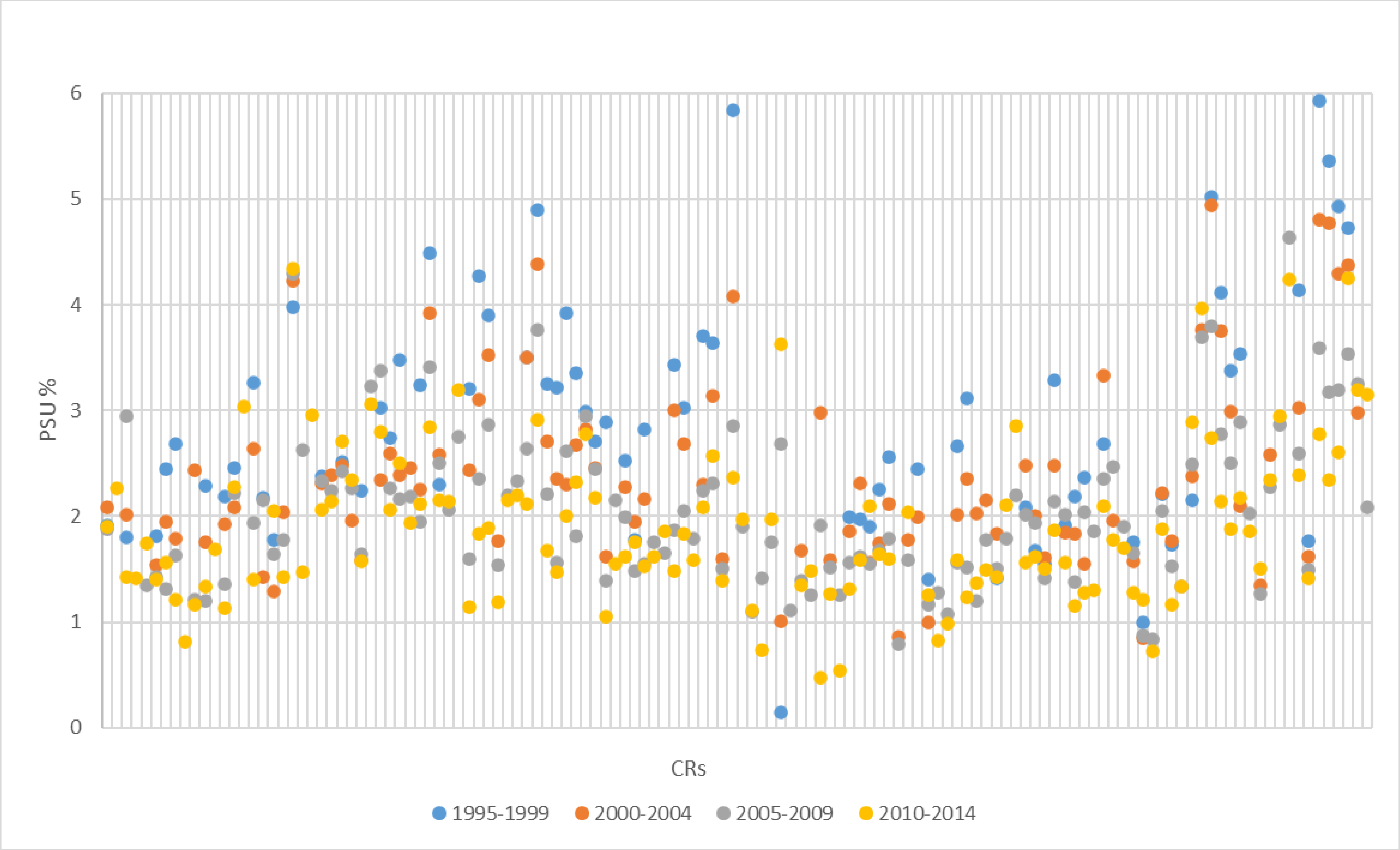


Supplementary Figure 7. Proportion of cases with unknown primary site/primary site uncertain (PSU%) percentage by period of diagnosis and PBCR, 1995-2014

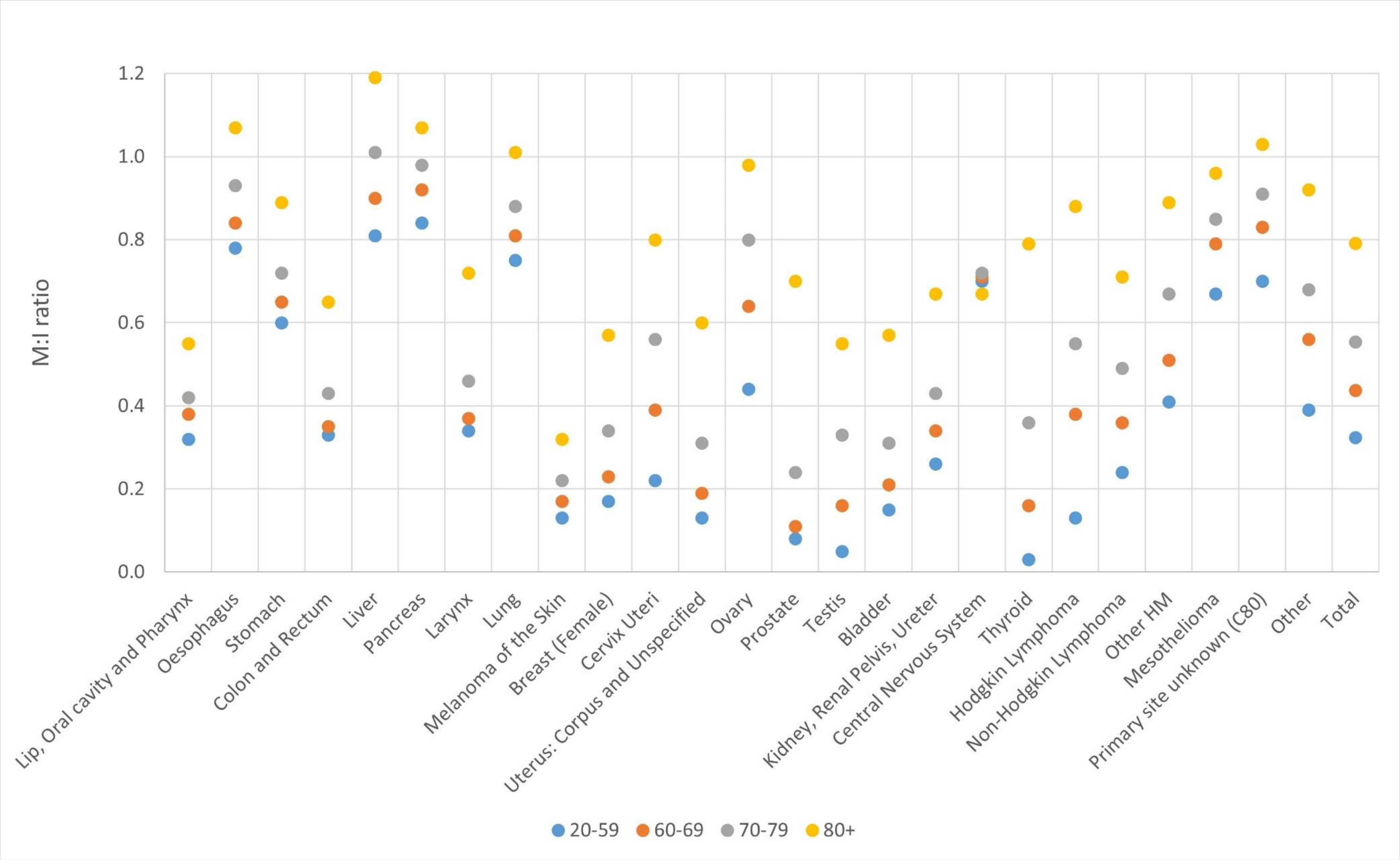


Supplementary Figure 8. Mortality to incidence (M:I) ratio by age at diagnosis and cancer entity, 1995-2014

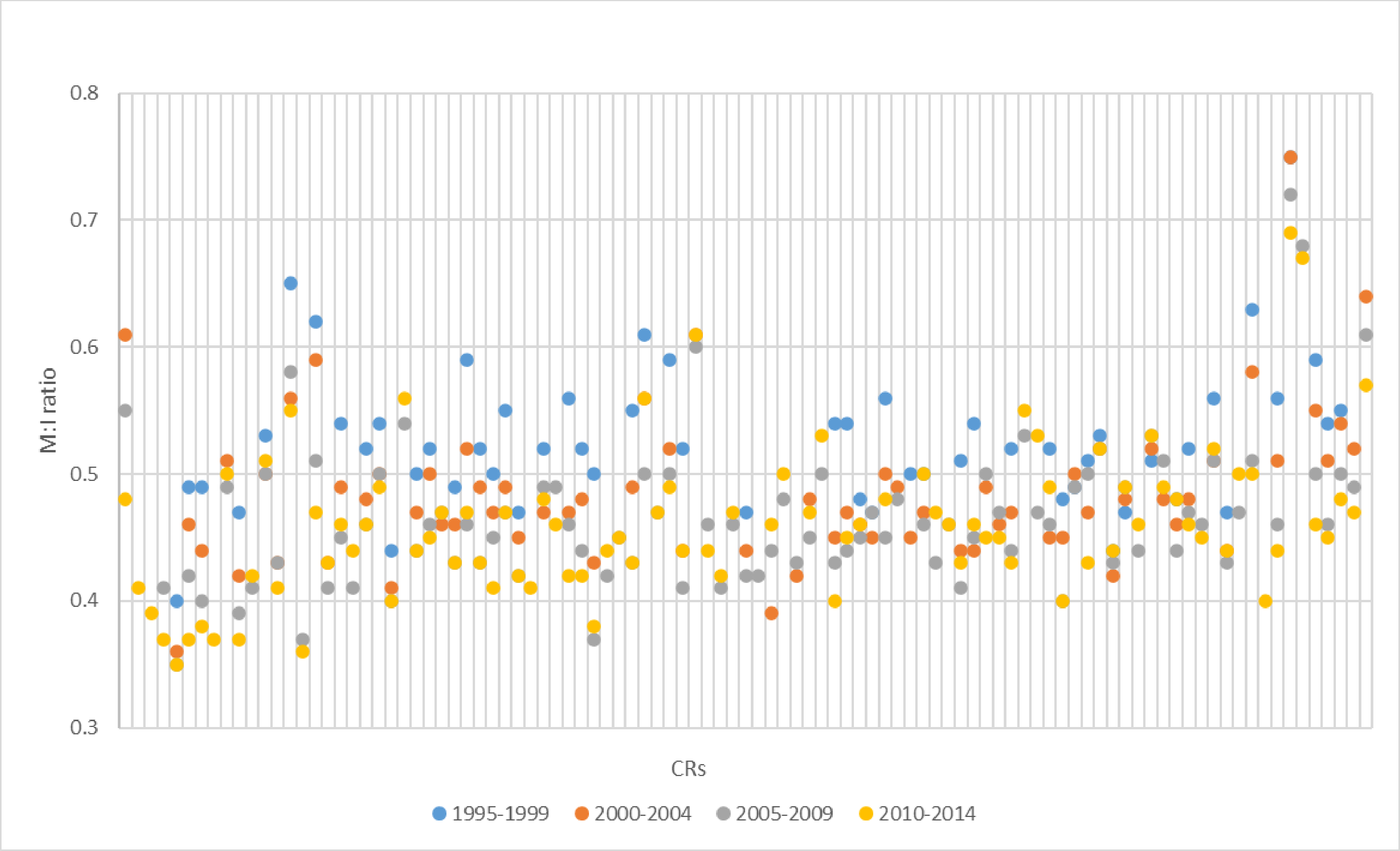


Supplementary Figure 9. Mortality to incidence (M:I) ratio by period of diagnosis and PBCR, 1995-2014

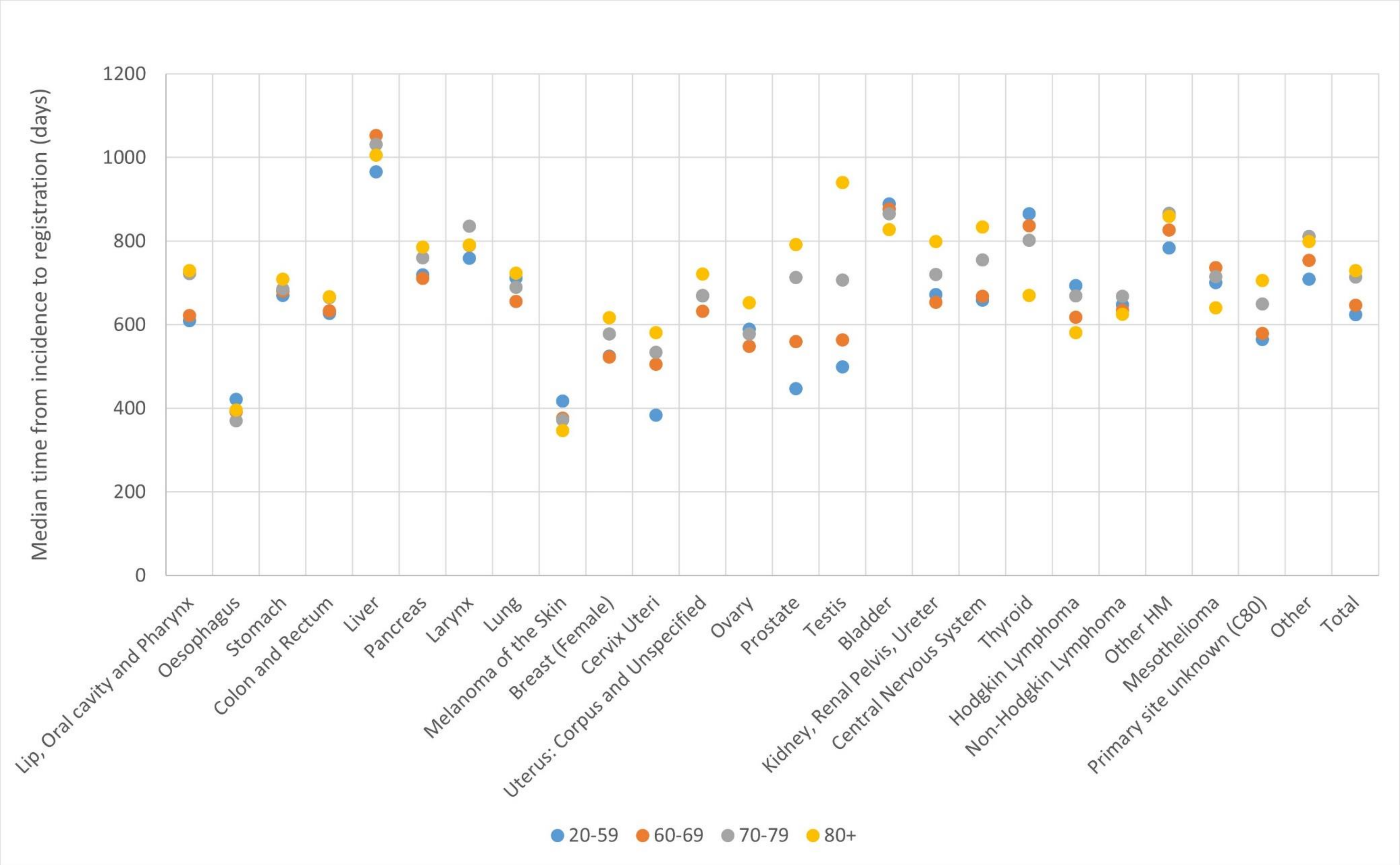


Supplementary Figure 10. Timeliness by age at diagnosis and cancer entity, 2000-2014

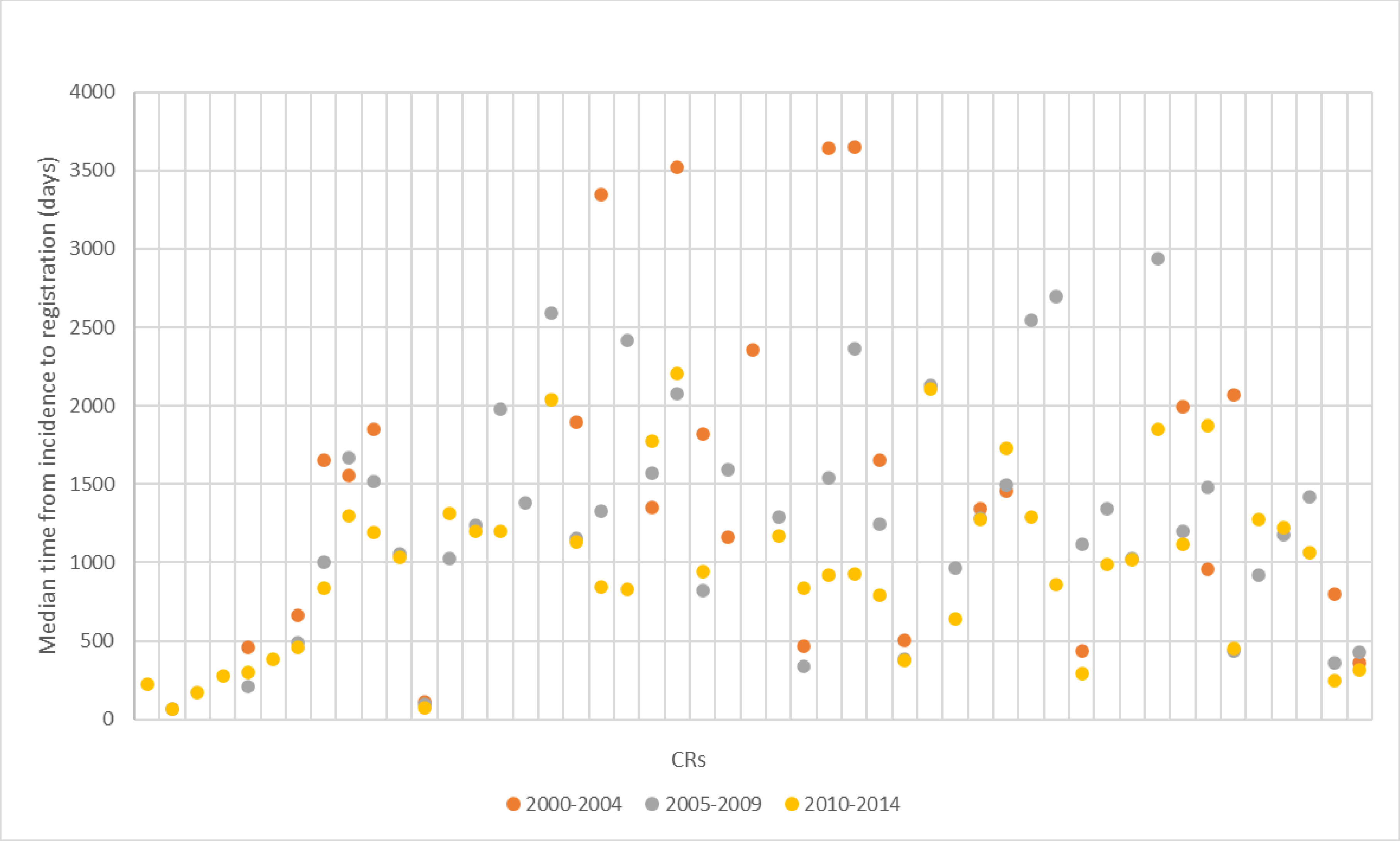


Supplementary Figure 11. Timeliness by period of diagnosis and PBCR, 2000-2014

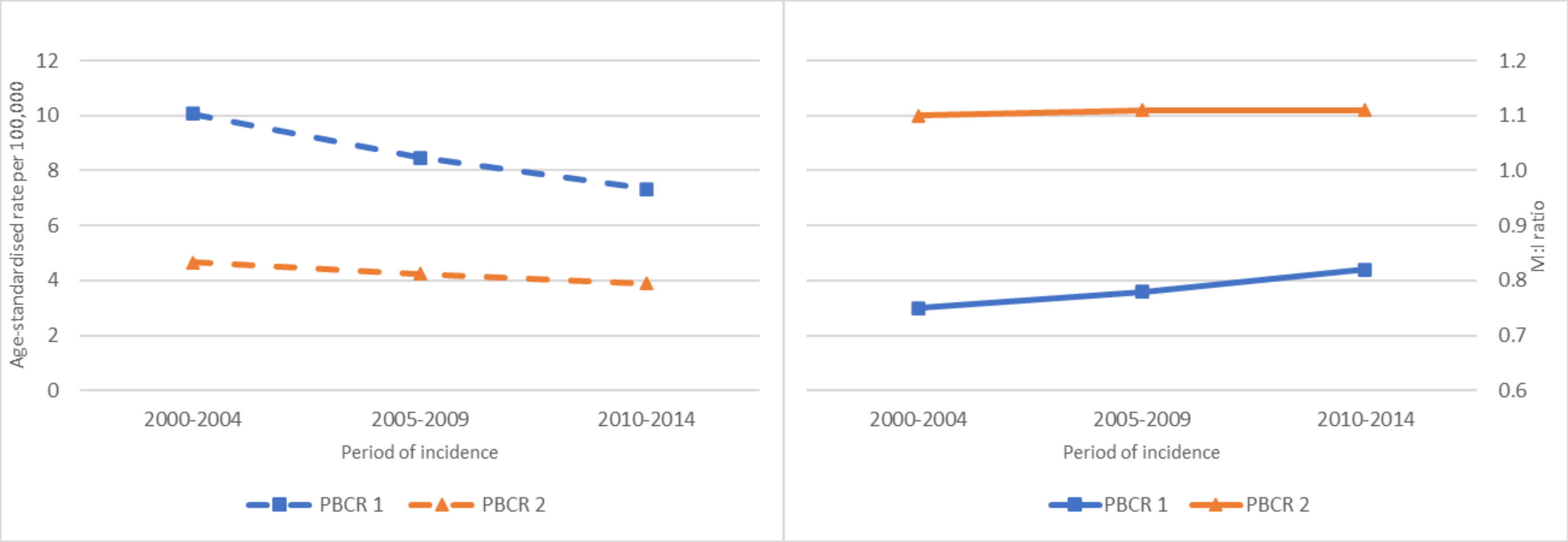


Supplementary Figure 12. Oesophageal cancer incidence (left) and M:I ratio (right) for two selected PBCRs, 2000-2014. PBCR 1 has higher incidence of oesophageal cancer and a lower M:I ratio than PCBR 2.
